# Comparing independent microarray studies: the case of human embryonic stem cells

**DOI:** 10.1186/1471-2164-6-99

**Published:** 2005-07-22

**Authors:** Mayte Suárez-Fariñas, Scott Noggle, Michael Heke, Ali Hemmati-Brivanlou, Marcelo O Magnasco

**Affiliations:** 1 Center for Studies in Physics and Biology, The Rockefeller University. 1230 York Ave, Box 212, New York, NY 10021, U.S.A; 2 Department Laboratory of Molecular Vertebrate Embryology, Camridge, The Rockefeller University. 1230 York Ave, Box 32, New York, NY 10021, U.S.A

## Abstract

**Background:**

Microarray studies of the same phenomenon in different labs often appear at variance because the published lists of regulated transcripts have disproportionately small intersections. We demonstrate that comparing studies by intersecting lists in this manner is methodologically flawed by reanalyzing three studies of the molecular signature of "stemness" in human embryonic stem cells. There are only 7 genes common to all three published lists, suggesting disagreement.

**Results:**

Carefully reanalyzing all three together from the raw data we detect 111 genes upregulated and 95 downregulated in all three studies. The upregulated list was subject to rtRTPCR analysis and 75% of the genes were confirmed.

**Conclusion:**

Our findings show that the three studies have a substantial core of common genes, which is missed if only the published lists are examined. Combined analysis of multiple experiments can be a powerful way to distil coherent conclusions.

## Background

A grave concern in the use of microarray technology is the apparently little agreement between different studies of the same phenomenon carried out in different laboratories, or even in the same lab using different platforms. Particularly evident is the "small intersection" problem: when several competing studies each conclude with a large list of "statistically significant" genes – yet the intersection between the published lists is ridiculously small. This problem undermines confidence in the technology[1] or, potentially worse, may misdirect the researchers into suspecting strange biological processes. When the subject matter is itself under contention then controversies may erupt [2-6]. The repeatability of microarray experiments across labs and platforms has become a hot current topic of discussion in the microarray community, with some studies suggesting that reproducibility across platforms is poor [7-9] while other studies indicate that biological and laboratory variability are larger sources of discrepancy than platforms or technologies [10-13], and with other studies pointing out annotations can be a source of problems[14].

We set out to study whether indeed different studies cannot be reconciled or whether the small intersection problem is artifactual, for one concrete problem of great scientific and extra-scientific importance: human embryonic stem cells (HESC). Isolation of HESC lines has generated the exciting possibility of both access to the basic science of human development as well as the possibility of new hope for cell-based therapy in the clinic[15]. The realization of these clinical goals depends on an understanding of the molecular basis of signaling pathways that maintain ESCs in an undifferentiated, pluripotent state, and microarray studies of HESCs should provide a sound basis for discovery and exploration of these pathways.

We shall consider 3 microarray studies of HESCs: Battacharya[16], Sperger[17] and Sato[18]. These studies had considerable differences, both in platforms (See Table 1) as well as biological. The Bhattacharya and Sperger studies have none or few replicates, hybridizing instead different cell lines to each chip, and contrast to different control samples of pools of differentiated tissues. The Sato study analyzed triplicates of a single cell line compared against "nonlineage induced differentiation" of that same cell line. Maintenance conditions also varied; see Methods for more details.

**Table 1:** Summary of platforms

	**Bhattacharya**	**Sperger**	**Sato**
Platform	cDNA -	cDNA – 12 × 4	Affy – HGU133a
Chip design	8 × 4, 23 × 23	12 × 4, 30 × 30	650 × 650
Scanner	GenePix 4000B	GenePix 4000	GeneArray
Image Analysis Software	GenePix 3.0	Gene Pix 3.0	MAS Suite
Spot/probes (per chip)	16 928	43200 / 43008	22 283
UniGene	12041	25 400	12 441
Empty spots per chip	238	531	-

Each study concluded with a list of genes which are up-regulated in stem cells. These lists are important because they should show a good view of all pathways in the cell for which there might be important stem-cell-specific functions, not just developmental signaling pathways, but also potential stem-cell-specific "housekeeping" genes. Most studies have a tendency to focus on developmentally important pathways, such as those that are sufficient for self-renewal; a genome-wide search giving us an unbiased list of up-regulated transcripts is supposed to give us a wider view into the state of stemness.

**Figure 1:**
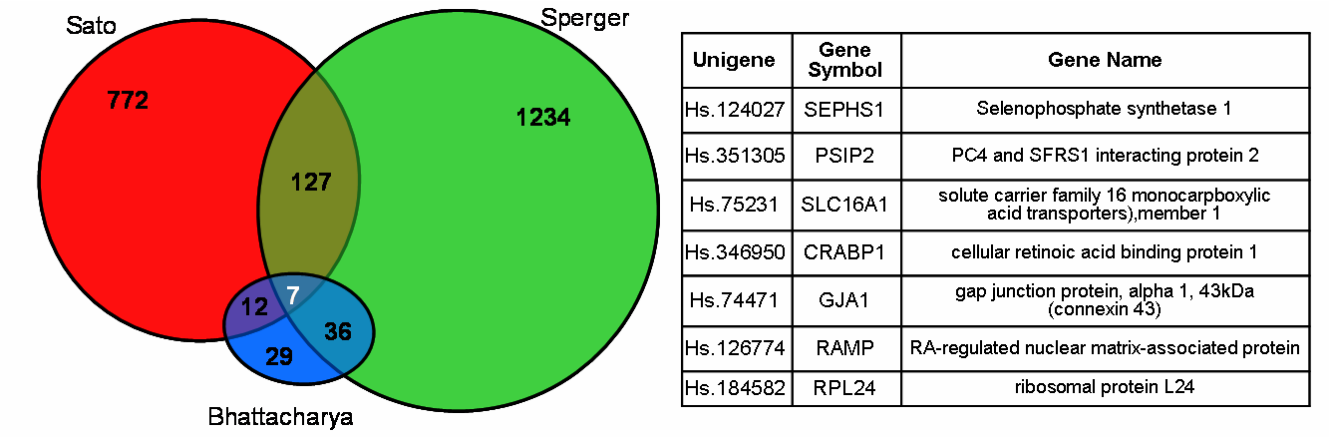
**Common published genes**. Intersection (per UniGene) between the published lists of up-regulated genes for each study.

But the three lists of significantly up-regulated genes, as published, are quite different from each other. Their intersection is shown in Figure 1: seven genes appear in all three studies out of 2226 total genes in the union of the three published lists. This is particularly troublesome since all three studies appear to be technically reliable and each study has good reproducibility between replicates (see Methods: "Within platform variations"). The intersection highlights mainly metabolic or housekeeping genes; significantly, few of these have been implicated in any of the nine major developmentally important signaling pathways.

The small intersection is a big problem: it is generally taken to mean that the results are not reproducible from laboratory to laboratory and should not be believed. We shall show that this is not the case, that comparing the lists in this manner is methodologically flawed and that the experiments do have a large core of common genes.

No experimental scientist would be surprised when results change upon a change of protocol; the analysis of microarray data, from image analysis to the statistical procedure, has as many parameters as a biochemical protocol, and hence there's little surprise that lists obtained by widely different "statistical protocols" are incompatible, so we shall now set about the task of defining and applying a single such statistical protocol to these studies.

## Results

In an experiment addressing differential gene expression, a common criterion to choose a p-value is the largest tolerable amount of false positives; then the power of the test to detect the true positives will depend on the difference between condition and control, the replication variability, the number of replicates and other parameters. In order to be at the intersection between three lists, a gene must have passed such a test three independent times, which it does with a probability equal to the product of the probabilities of each test. Therefore the intersection's p-value is the cube of the lists' p-value. This strongly throws off the balance between sensitivity and specificity, with the result that the intersection becomes insensitive and carries few genes (See section D1 of Additional file 1 – Supplementary Discussion and Methods.). The lists which are good for publishing are inadequate for intersecting; in order to generate the best intersection the lists have to be recomputed.

We procured the raw data from all three studies, and re-analyzed it using consistent criteria for spot quality, normalization and summarization, in order to obtain the expression measures for the three studies. Since all three platforms are different (see Methods: "description of studies") they have many genes which are not in common and so could not possibly be at the intersection. We carefully screened UniGene numbers to obtain the set of 7373 genes common to all 3 microarray platforms, which is the "universe" of our study. Most of these genes show variations between stem lineage and differentiated control which are no larger than replication errors.

Eliminating them increases the power of our analysis and thus we kept for further study only those 2463 genes in this set that displayed variations across the samples, since only these could be differentially expressed genes. It should be emphasized that our universe does not include all of the genes possibly involved in stemness. For example some genes, like TDGF1 (Hs385870), Nanog (Hs329296), DNMT3B (Hs252613), FOXD3 (Hs424212), OTX2 (Hs288655), MyosinX (Hs481720), HEY2 (Hs144287), FGF4 (Hs1755), Rex1 (Hs335787) and Nodal (Hs370414), most of which were highlighted as up-regulated in HESCs in various studies, were not included in our "universe" because they are missing from at least one of the three different chip designs.

We then analyzed the genes by Integrated Correlation Analysis[19] (see Methods), which was introduced to validate the agreement among studies and to select genes that exhibit a coherent behavior across different studies. The idea is that while studying the same system, co-regulated genes should exhibit correlated expression profiles, and these correlations should be maintained across studies. This quality of moving together with other genes we call "coherence". Conversely, when extraneous factors affect a small set of genes in a particular study, the correlations between those genes and the rest shall not be maintained across studies.

**Figure 2:**
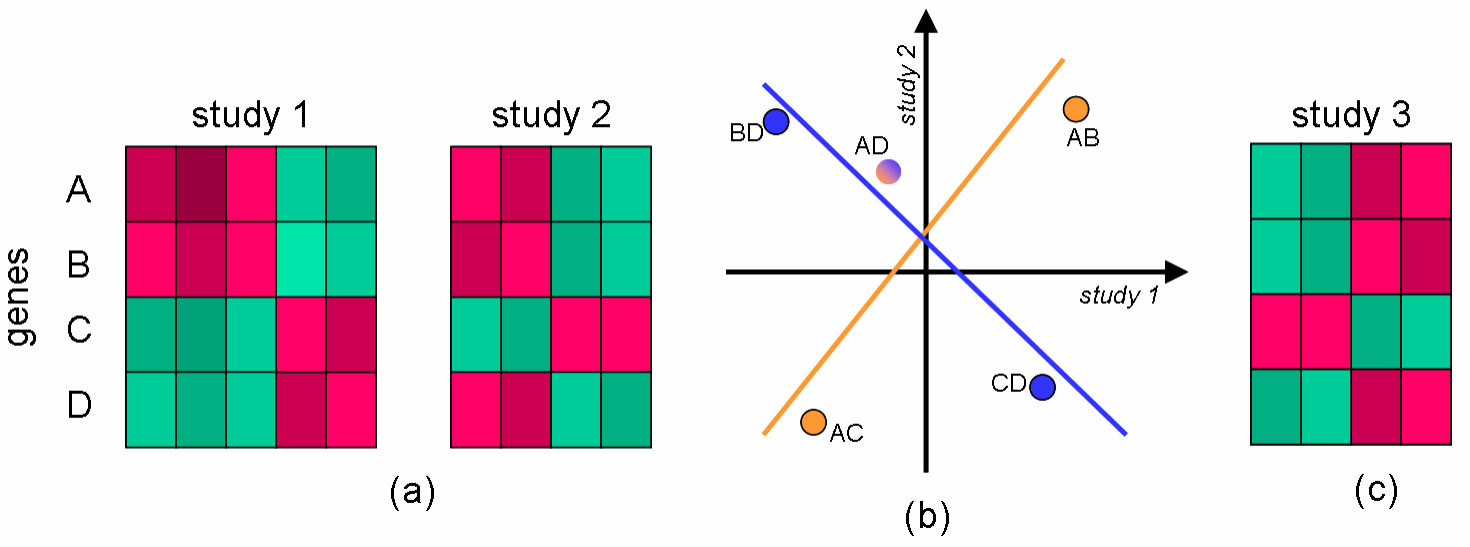
**Coherence Scores**. Given the expression profile for four genes A, B, C, and D in two studies 1 and 2 shown in panel (a), we can calculate the correlation of the expression profiles for every pair of genes in each of the two studies. In panel (b) those pairwise correlations are plotted for study 1 against study 2. For example, pair AB is positively correlated in both studies and pair BD is negatively correlated in study 1 and positively in study 2. Correlations for pairs AB, AC, AD lie approximately on a line of positive slope, so gene A is called "coherent". The coherence score of A is the correlation coefficient of those points, which is positive. Gene D has a negative correlation coefficient and so is incoherent. Study 3 in panel (c) is study 2 where condition and control have been swapped; all genes are perfectly coherent for studies 2 and 3, yet each gene which is up-regulated in study 2 is down-regulated in study 3.

Figure 2 illustrates how "coherence" of genes between two studies is quantified. For a pair of genes, the correlations between their expression profiles are calculated within each study and the correlation in one study is plotted against the other; points near the identity line represent pairs of genes which maintain their correlation values across studies. The coherence score of a gene *g *is the correlation coefficient of the points corresponding to all pairs containing *g*. Using all gene pairs we obtain the integrated correlation score, an overall measurement of agreement between the two studies. We have changed the nomenclature from [19], where coherence is called "consistency", because we want to reserve the notion of consistency to mean moving in the same direction from condition to control in all studies (i.e., up-regulated in all studies). Notice that no information about the condition and control is introduced in the definition of coherence; if the genes are both up-regulated in study 1 and both down-regulated in study 2 they are coherent because they behave the same way with respect to each other within each study, even though they are both inconsistent. (See Methods: Coherence and Consistency)

**Figure 3:**
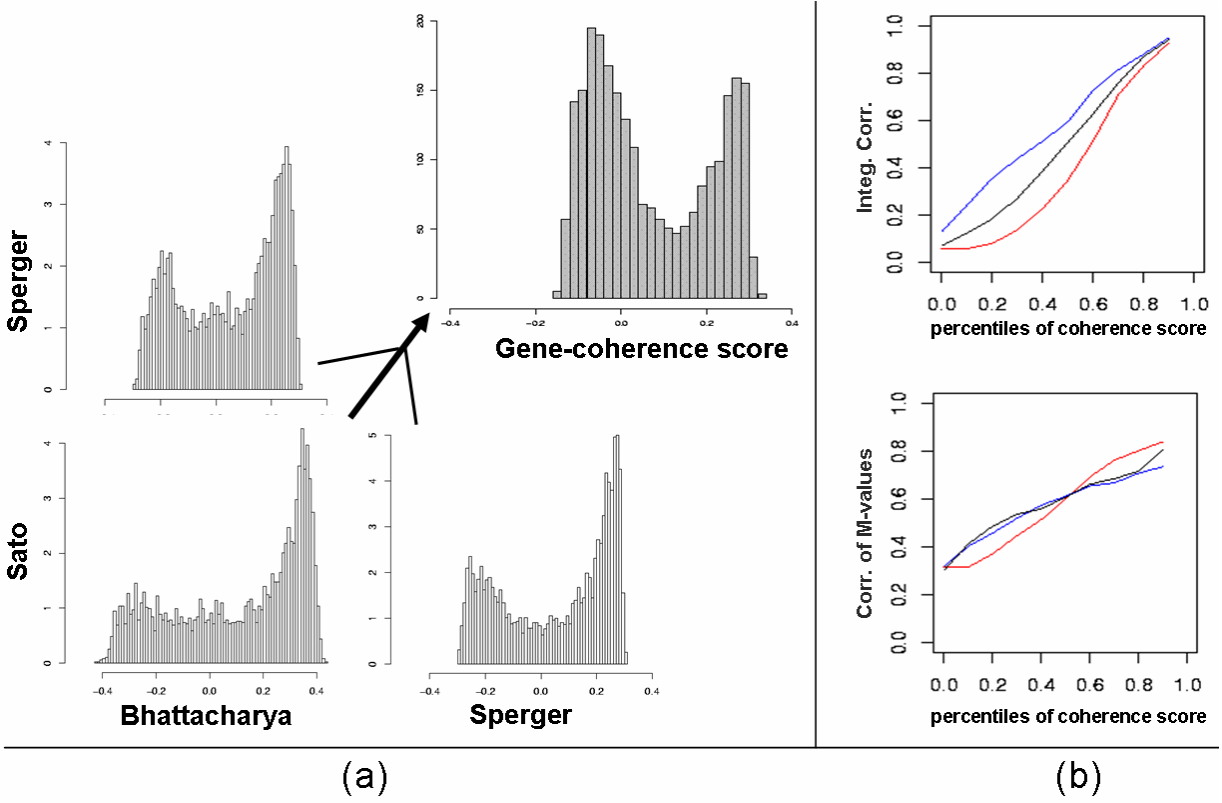
**Coherence Scores Distribution**. The histograms of the coherence scores are bimodal. (a) Pairwise comparisons, and averaged score over all three comparisons. This implies that in these studies the genes can be divided into two distinct categories, coherent and incoherent. When "erratic" genes are discarded, there is a marked improvement in the agreement between studies. b) Integrated Correlation and Correlation of M-values calculated using the genes in the top percentiles of Coherence Score, red: Bhattacharya-Sperger, blue: Bhattacharya-Sato, black: Sperger-Sato

Previous use of this analysis applied to classification of cancer [19] yielded unimodal histograms of the gene-coherence scores (there called "gene-reproducibility score"); in our study, though, we obtain a strongly bimodal histogram, shown in Figure 3, indicating that there is a number of genes in strong agreement among the three studies and a number of genes in strong disagreement. (Further details, including bivariate density plots, can be found in Additional file 1, section D4). There are a number of potential reasons why genes could be strongly incoherent as shown in these histograms discussed later.

Eliminating incoherent genes improves enormously the general agreement between the studies. The improvement in the integrated correlation score and the correlation between log_2_-fold changes (M-values) is illustrated in Figure 3b. We decided to keep for further analysis the 739 genes in the top 30% of the gene-coherence score distribution. (Little variation of our final results was observed for percentiles 40% and 50% since they only include positive coherence scores; we present the stricter criterion). Not so obviously, the correlation between the M-values between studies also markedly improved from 0.35 to 0.76, 0.68, and 0.66 respectively. (More detailed results about Integrated Correlations results can be found in Additional file 1, section D4)

Once we restrict ourselves to this set of coherent genes, we study those genes that are up- or down- regulated in stem cells vs. their differentiated controls in each one of the studies. We emphasize that exactly the same statistical tests and criteria were applied to all three studies, with a strict cutoff value selection based both on a p-value and a positive lods [20]. We used the moderated t-statistics as proposed by [21] and the FDR (BH) procedure was used to adjust the p-values for multiple hypothesis [22]. This is important because the set of coherent genes is enriched in genes that are not statistically independent. The BH procedure [23] controls FDR under certain general assumptions (positive regression dependence) and simulations shows its adequacy to control FDR in more general dependence structures [24], while the BY procedure [25] is assumption-free but is more conservative that BH. However, in our case both procedures led to practically the same results, because the proportion of differentially expressed genes is higher in the coherent set; we report the results from the BH. P-value cutoff was set in 0.01, which implies than the probability of error is 10^-4 ^in the pairwise comparison and 10^-6 ^when the three studies are considered.

**Figure 4:**
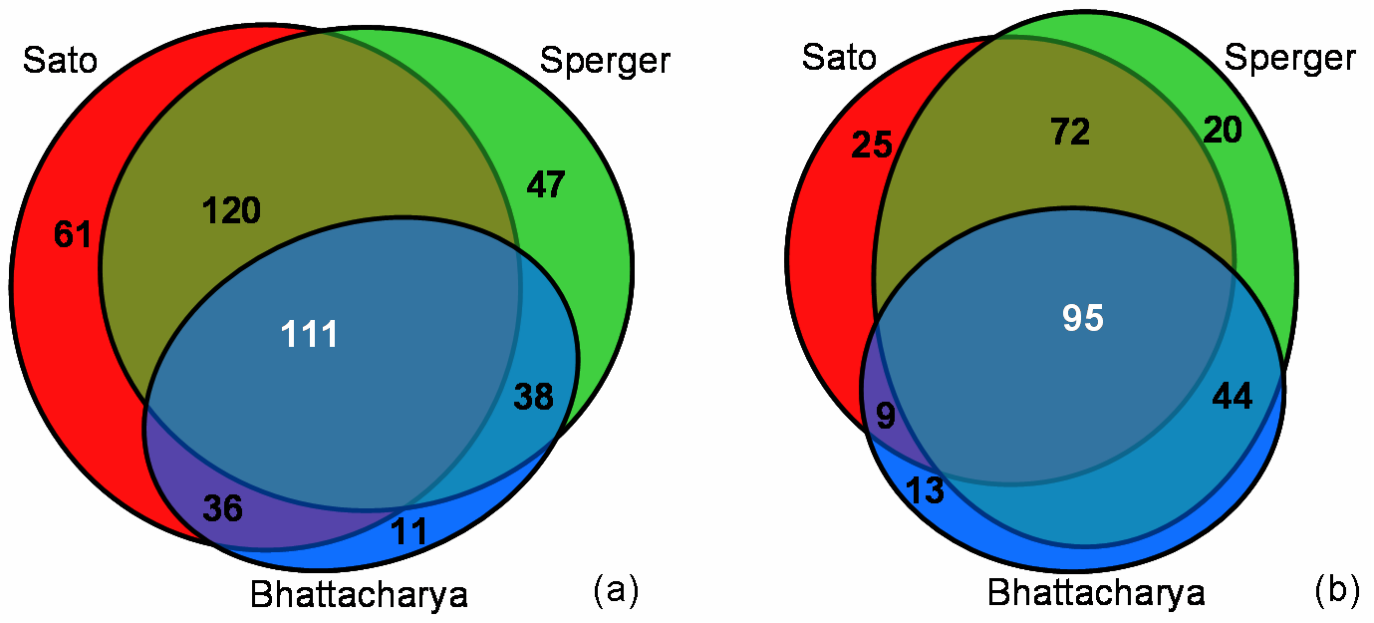
**Intersection of significant genes**. Improved intersection of significant genes. After our screening process, the number of transcripts which are up (a) and down (b) regulated in stem cells for the three studies. Notice that now most genes in each study also appear at least in one other study (81% Bhattacharya, 85% Sperger, 94% Sato, about 11% expected by chance), with a very important fraction common to all three (about 3 genes expected by chance at the intersection).

The intersection between the lists is now quite larger and statistically significant, as shown in Figure 4; the 111 up-regulated and 95 down-regulated genes common to all three studies (vs. 3 expected by chance intersections) are listed in Additional file 2 and Additional file 3 online. Notice that the 111 up-regulated genes in this list are not necessarily the "most" up-regulated for any individual study; yet they are significantly up-regulated for each study.

We performed real-time RT-PCR analysis of the up-regulated genes to validate our findings. In an initial experiment, H1 HESCs were differentiated for 30 days with RNA samples taken before and after differentiation. We succeeded in analyzing 106 genes out of the 111 intersecting HESC enriched genes in triplicate for expression level changes upon differentiation by real-time RT-PCR (See Additional file 4 for primers used in real-time RT-PCR). After screening for significant differences (within this experiment) between the mean normalized Ct values (Student's t-test, p > 0.05, n = 3), between differentiated and undifferentiated samples, 87 genes (82%) had differences in expression. Of these, 77 (89%) were enriched in undifferentiated HESCs and 10 (11%) were enriched in differentiated HESCs. Overall, 88 of the 106 tested genes (83%) were enriched in undifferentiated HESCs (p = 1.5 × 10^-12 ^in the exact binomial test); 77 of them also significant by RT-PCR, for a 73% of success probability (p = 1.7 × 10^-6^). 9% were enriched in the differentiated sample and 18% were either not regulated or not significant in this experiment. (See Additional file 5 for the raw data of real-time RT-PCR results)

**Figure 5:**
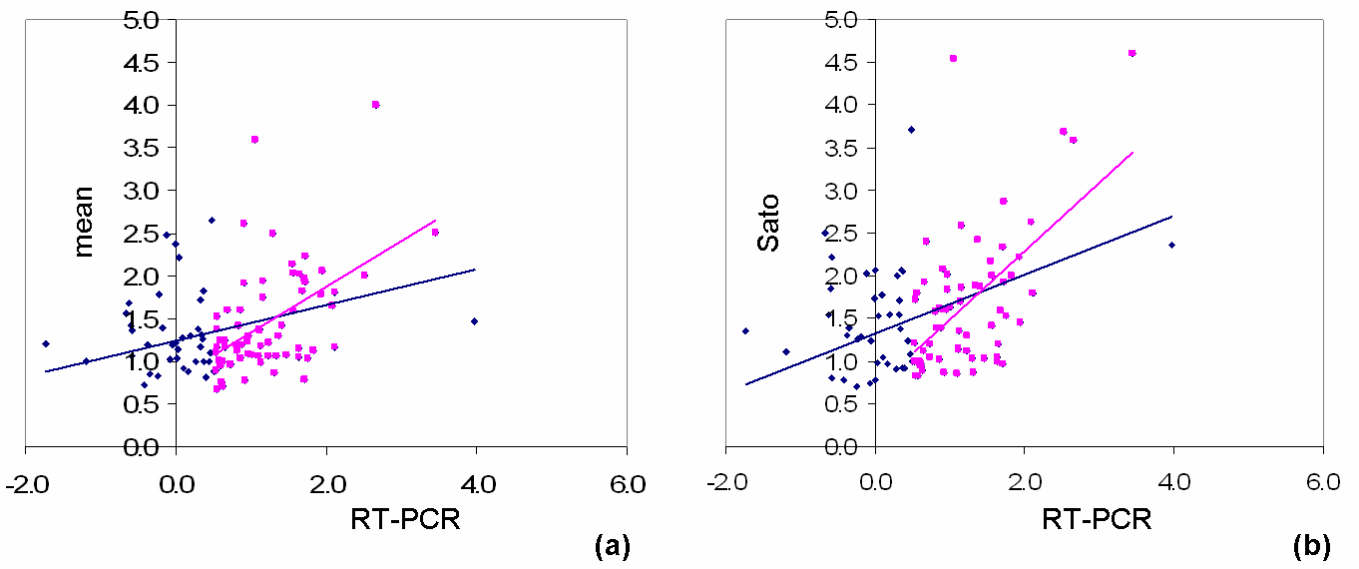
**RT-PCR results**. Comparison between the real-time RT-PCR results and the microarray results. The blue lines are linear fits (without intercept) through all the 106 genes, while the magenta lines fit only the 67 genes with a fold change bigger than 0.5 (log2-scale) in RT-PCR analysis (magenta points). (a) log2-fold change of RT-PCR vs. mean of all microarray studies (R^2 ^= 0.5, r = 0.32, p < 10^-16 ^for all genes, R^2 ^= 0.83, r = 0.51, for the 67 top genes); (b) log2-fold change RT-PCR vs. Sato study alone, which had identical conditions to our study (R^2 ^= 0.53, r = 0.41, p < 10^-16 ^for all genes, R^2 ^= 0.85, r = 0.59 for the 67 top genes)

A comparison between the fold changes found through real-time RT-PCR and those obtained in the three studies is shown in Figure 5. Our real-time RT-PCR test was carried out under identical cell line, culture and differentiation conditions to the Sato study, so we present comparisons against the mean log-fold-change of the three studies and against Sato's study separately, which has for evident reasons better fit. Positive slopes and correlation coefficients are obtained in all comparisons; correlations are higher when fitting only to genes with real-time RT-PCR log2 fold-changes bigger than 0.5, becoming 0.6 when comparing only to Sato's study. These slopes and correlation coefficients are in line with general results in the literature comparing different microarray platforms and real-time RT-PCR results for the same RNA sample.

## Discussion

How can we reconcile the agreement in Figure 4 with the disagreement in Figure 1? Figure 4 displays an incorrect comparison, since intersecting lists in this manner is flawed methodology. The three lists were created from different sets of genes using different statistical criteria for ranking the transcripts; they really are apples and oranges and should not be compared so lightly. To mention but three exemplars of problems with such comparison:

- there are more than 40000 genes in Sperger's study, yet only 7373 are common to all three arrays, so about 4/5 of the genes studied by Sperger were not studied in some other study; in fact, more than 650 genes in the published list could not possibly have been at the intersection.

- the size of the intersection cannot be larger than the smallest list and is thus controlled by it; in this case the Bhattacharya study had far more stringent criteria and reported the fewest genes; notice that about 2/3rds of that list were in at least one of the other studies.

- different versions of annotation databases were used to create the lists, so some genes have identifiers that changed over time and are missed in an automated comparison; in fact, when a current version of the Affymetrix probeset annotations is used for the Sato study, the intersection increases more than twofold to 16 due to several genes having changed identifiers

These are just exemplars of the general problems of differences in gene universe (a), in gene identity (c), and in statistical criteria (b), which include not only the significance threshold used, but which particular test was employed to assess it (e.g. "moderated t-statistics with fdr corrections for multiple hypothesis"). Other general problems affecting list intersection also include the preprocessing steps, which have been shown to have a substantial impact in the agreement between platforms[11,14]. So, how should different experiments be compared? Treating them on an equal footing goes most of the way: this means procuring from the authors the raw data, analyzing all studies with exactly the same statistical methods and cutoffs, and working only on the universe of common genes. This still does not make the studies all apples, but it significantly approaches this goal. We further refined this by using the notion of gene coherence; in doing so we discovered that a number of genes are strongly incoherent, behaving erratically across the studies.

The incoherent genes may behave so due to environmental interactions, differences in cell culture system or in the differentiated states being compared. Gene expression is the nervous system of cells, imprinted by anything in the environment. A large number of genes are involved in environmental interaction, leading to variability between labs which obscure the common signal we are trying to detect. Among the gene categories that classify the top 50 incoherent genes (see Additional file 6), i.e. those that exhibited a significant fold change in respective studies but that were regulated in opposite directions among the studies, the top classifications were mainly involved with metabolic activity, such as genes involved in the response to oxidative stress and reactive oxygen specific genes. The incoherent genes may also reflect differences in the conditions that the labs use to grow cells. The HESC culture system has many poorly understood variables, such as batch to batch variation in the MEFs used to support the HESCs and the use of serum in some culture systems. These are both examples for which there are, as yet, no good predictors for the ability to support "stemness" and can currently only be screened by their ability to support HESC maintenance based on few markers. However the list also includes developmentally important genes, such as MID1(Hs27695), a possible left-right determination factor [26], and FGF9(Hs111), implicated in several developmental decisions. Also included were several members of the Wnt pathway, including APC(Hs158932), DIXDC1(Hs116796), and TLE4(Hs444213), so the incoherent genes may also reflect differences in the control samples used in the studies. The Sato group uses HESCs differentiated by withdrawal of CM, which may result in biases in the differentiated cell types produced, while the other groups use pooled panels of RNA from a variety of somatic tissues.

The application of microarray technology to the study of HESC biology has the potential to provide a robust foundation for the exploration of molecular networks underlying the state of "stemness" in human embryonic stem cells. In addition to the insight into their utility in clinical applications provided by this exploration, elucidation of these networks in HESCs shall be an important step towards defining the molecular activity driving development in early human embryos.

Our list of confirmed transcripts intersects three experiments carried out on different cell lines, maintained in the stemness state by different protocols, and compared against different differentiated states. That we do get a confirmed list under these circumstances bears witness that there is a well-defined set of molecular circuits involved in the state of stemness that can be studied regardless of variations in protocol or cell line. This analysis more than octuples the list of confirmed molecular markers of the stemness state in HESCs [27].

A balanced global assay of the molecular state of "stemness" should reflect all activities of the cell including those pathways that are necessary as well as those that are sufficient for the functions of an embryonic stem cell. Therefore, we should expect to find activities that might include, for example, those required for the defense of chromosomal or genetic stability as well as those required for self-renewal in an undifferentiated state. Indeed, the two most represented GO Biological Process in a GO/EASE analysis of the enriched genes within our universe are "Traversing start control point of mitotic cell cycle", indicating a prominent role of cell cycle regulators, and "transmembrane receptor protein serine/threonine kinase signaling", indicating a significant role for genes involved in sensing and responding to the developmental environment. Further, "transforming growth factor-beta receptor activity" was the most represented GO Molecular Function classification, indicating a prominent role for TGF-beta signaling in undifferentiated HESCs (See Additional file 7 and Additional file 8 for the complete GO analysis). However, because many of the genes that are important for these and other activities may be excluded from the universe analyzed here, we do not present the results as a complete or unbiased picture of these activities. Instead, this study addresses the validity of using microarray technology for building this foundation by applying consistent analysis to evaluate reproducibility.

## Conclusion

Our study supports concluding that the three studies are compatible and repeatable. The method we've used demonstrates that we can harness the power of several labs to give weight to the intersection. In fact, we may conclude that a list of regulated transcripts from a single lab obtained under a restricted number of maintenance and differentiation protocols should be considered with some reserve, for we only know about coherent behavior of genes when we have several studies to draw from. Our results indicate that publication of the raw data is far more valuable than publication of the analyzed data, and further suggest that the field shall move forth only upon agreement to set standards of backgrounds and statistical methods.

## Methods

(see Additional file 1, section Methods for a full description)

### Description of the studies

The Bhattacharya study has 6 chips. Different HESC lines were hybridized to the red channel (Cy5) of the arrays; 5 of them were lines BG01, BG02, GE01, GE09, TE06, and the sixth sample was a pool of GE01, GE07, and GE09. The control sample, hybridized to the green channel was "total human universal RNA (huURNA) isolated from a collection of adult human tissues to represent a broad range of expressed genes from both male and female donors (BD Biosciences, Palo Alto, CA)". No replicates were performed for individual lines. The Sperger study used a similar design, hybridizing lines H1, H7, H13, H14 and two samples of H9. The control samples were "a common reference pool of mRNA". The Sato study had 6 Affymetryix HGU133A chips, 3 replicates of H1 cells (in Matrigel/Conditioned Medium) and 3 replicates of "nonlineage-directed differentiation" (Matrigel/non-CM). Table 1 summarizes the architecture of chips and the number of spot/genes involved in the three studies.

### Language and packages

The statistical analysis was carried out in the R language version 2.0 , and packages were from the Bioconductor project . Gene Ontology analysis was carried out using EASE 2.0 software [28].

### Raw data

Data from the Bhattacharya and Sato studies were obtained directly from the authors. The Sperger data were obtained through the Stanford Microarray Database [29]. cDNA array data were output files from GenePix 3.0. Affymetrix raw data files were .CEL files.

We used the same image analysis criteria in all CDNA arrays to exclude low quality spots (the criteria were different in the published studies).

### Expression measures

The marray package from the Bioconductor suite was used for cDNA arrays. Normalization was executed in two steps, first within-print-tip-group location-dependent intensity normalization followed by within-print-tip group scale normalization using median absolute deviation. Single-channel normalization of two-color cDNA was done as proposed by [30], using quantile normalization. The GCRMA algorithm was used to summarize Affymetrix data as proposed in [31]. This algorithm improves the widely used RMA [32] by including an extra step to adjust for non-specific binding, and computing the sequence-specific affinities between probes as described [33].

### Within-platform variability

In order to assess the quality of the data replications the within-platform variations were analyzed. The within-study reproducibility is overall fairly good in all the studies, even noting that Bhattacharya's and Sperger's design contain different lines of HESC rather than true replicates of a single line. See further details in Additional file 1.

### Annotations

For both Bhattacharya and Sperger studies, annotations were obtained from SOURCE from the Stanford microarray data homepage[34]. For Affymetrix data, annotations packages from Bioconductor were used. The IMAGE clone IDs and the Affymetrix probes were matched using UniGene Cluster Annotation. Genes with no UniGene number were eliminated from the study. Expression values from spots or probesets with duplicated UniGene identifiers were averaged together.

### Common genes

Annotations were obtained with the raw data from each study. Genes without UniGene identifier were eliminated and duplicated probes/spots were averaged together. After this process there are 7373 genes common to all 3 studies as shown in Supplementary Discussion Figure 8a. We filtered for evidence of variation across samples, reducing our set of interesting genes to those showed in Supplementary Discussion Figure 8b. For the cDNA arrays, we select genes where the M-values was bigger than 0.3 in at least 4 arrays and in Affymetrix experiment we keep genes whose expression profile had range bigger than 0.5.

### Coherent genes: the integrated correlation approach

Integrated correlation analysis was introduced in [19]. For each study s, let us define x_g _the expression profile for a gene g, and  the correlation for the pair of genes p = (g_1_, g_2_) in the study. Based on  we can asset both overall coherence between studies and gene-specific coherence. The integrated correlation, defined as:  quantified the coherence between studies. If this expression is calculated considering only the pairs containing a gene g, then we have a measure of the gene-specific coherence between two studies: , where p = (g, j) When more that two studies are involved, the average over all s and s' is used as a Coherence Score for a gene g, . Confidence Interval for the correlation scores were obtained by bootstrapping.

### Coherence and consistency

In [19], the coherence score defined above is called reproducibility score and coherent genes (genes with high values of the score) are called "consistent". However we once again stress that this score bears no direct logical relationship to the notion of reproducibility or consistency in the sense of consistent up- or down- regulation in both studies. A simple counterexample makes the point: create a fake Study 3 which is Study 2 with the values of the condition and control swapped; then by the above definitions, all genes are perfectly coherent for studies 2 and 3, having coherence (reproducibility) scores equal to 1; yet each gene which is up-regulated in study 2 is down-regulated by the same amount in study 3, so all genes are inconsistent. A relationship between the coherence scores and consistent behaviour is predicated on the counter-reciprocal: if a pair of genes is incoherent then both genes cannot be consistent, and hence if a gene has a negative coherence (reproducibility) score it is "likely" (though by no means sure) to be inconsistent by being the "odd one out".

### Differentially expression criteria

Statistical analysis to determine which genes are differentially expressed was carried out using the package Limma from the Bioconductor project. For assessing differential expression the moderated t-statistics was used as proposed by [21] in all the 3 studies. To do so, Limma uses an empirical Bayes method to moderate the standard errors of the estimated log2-fold changes. This results in more stable inference and improved power, especially for experiments with small number of arrays. The p-values of the moderated t-test were adjusted for multiple hypothesis testing, controlling the false discovery rate (fdr) as proposed [22]. We use a strict cut off criterion for selectivity of the genes based on both the p-values and lods ratio, as proposed in [20]. The lods (or B-statistic) is the log of the odds that the gene is differentially expressed. For the set of coherent genes we selected here, the cutoff of p = 0.01 and positive lods leads to 206 (111 up, 92 down) genes while 244 (139 up 105 down) is obtained considered only the p-value cut-off. In Supplementary Discussion and Methods the reader can find how the number of selected genes depends on the criteria for different set of coherent genes.

### Real-time RT-PCR verification of gene expression level in HESCs

Relative expression levels of the 106 (95%) of 111 genes in the intersection were analyzed in H1 HESCs maintained in CM or differentiated by withdrawal of CM for 30 days by real-time RT-PCR. Raw data were normalized to Ubiquitin-C expression and relative expression levels were determined using PCR efficiency-adjusted ratios [35].

## Authors' contributions

MSF and MOM are the analytical members of the team while SN, MH, AHB are the biological team. Conception of the work came from join discussion between MSF and AHB. MSF and MOM are responsible for analysis and methodology. MH designed the primers for the RTPCR experiment, carried out by SN and AHB; the latter are also responsible for the biological assessment of all analytical results.

## Supplementary Material

Additional File 1 Supplementary Discussion and Methods. Click here for file

Additional File 2 **Up-regulated genes in the intersection**. List of up-regulated genes in the intersection of the 3 studies. In html format, including annotations and links. Click here for file

Additional File 3 **Down-regulated genes in the intersection**. List of down-regulated genes in the intersection of the 3 studies. In html format, including annotations and links. Click here for file

Additional File 4 **RT-PCR primer sequences used**. Primer sequences used in real-time RT-PCR. Click here for file

Additional File 5 real RT-PCR results. Click here for file

Additional File 6 **Top 50 incoherent genes**. List of the top 50 genes with the most negative coherence score. Click here for file

Additional File 7 **Up-regulated genes – EASE analysis**. EASE analysis for the up-regulated genes in the intersection of the three studies Click here for file

Additional File 8 **Down-regulated genes – EASE analysis**. EASE analysis for the up-regulated genes in the intersection of the three studies Click here for file
