# Supplementary material for: Comparing independent microarray studies: the case of human embryonic stem cells
Source: BMC Genomics. 2005 Jul 22;6:99. doi: 10.1186/1471-2164-6-99 (PMC1183205; doi:10.1186/1471-2164-6-99)
Supplement: Additional File 1 — Supplementary Discussion and Methods. [file 1471-2164-6-99-S1.doc]

How to compare microarray studies:

The case of human embryonic stem cells

Mayte Suárez-Fariñas*, Scott Noggle§, Michael Heke§, Ali Hemmati-Brivanlou§ and Marcelo O. Magnasco^*^

^*^Center for Studies in Physics and Biology and ^§^Laboratory of Molecular Vertebrate Embryology,The Rockefeller University. 1230 York Ave Box 212, New York, NY 10021, U.S.A.

# Supplementary Information

# Discussion

## Basic math of list intersection

Consider first an ideal situation in which the three experiments being compared are identical except for their replication errors. Each experiment has D genes up-regulated by an amount F, N genes which are not differentially expressed, condition and control have been replicated R times, and the replication errors are independent, identically distributed Gaussians with mean 0 and standard deviation ε. Let’s assume for example D=100, N=10000 (the size of the chip), F=2 (in log_2_ scale, i.e. fourfold), R=3 and ε =0.3 (in log_2_ scale, i.e., 22%).

For each experiment a list is created by applying a t-test, sorting the genes by the p-value of the test and cutting off the list at some threshold p_th_. For large p_th_ the list is sensitive (i.e., includes all the right genes) but nonspecific (has many false positives). As p_th_ is lowered and the list becomes more stringent, false positives are rapidly lost at the expense of losing some true positives, and if p_th_ is made too stringent the list becomes specific at the expense of becoming insensitive. Choosing the p-value controls the number of false positives (Type-I error); once fixed, the power of the test depends then on the sample size, the difference and p_th_.

To be at the intersection, a differentiated gene must be in each of the three individual lists, and so must have been correctly chosen three times; if the lists are statistically independent this happens with a probability equal to the product of the probabilities of choosing correctly in each list (called the true positive rate, tpr). Similarly, a nondifferentiated gene will be at the intersection only if it was incorrectly chosen all three times and occurs with a probability equal to the product of the false positive rates (fpr) of each list. The number of expected errors equals the rate times the number of genes in each corresponding class: the number of true positives TP is expected to be tpr*D; for the false positives, FP=fpr*N, and so the number of false positives is amplified relative to the true positives because there are so many more undifferentiated genes.

In other words, intersecting lists can be thought of as a statistical decision rule given as the logical conjunction of several tests. The tpr and fpr of this rule are the product of the tprs and fprs of the individual tests, and are therefore both smaller (and potentially *much* smaller) than the individual ones, so the intersection test is less sensitive and more specific.


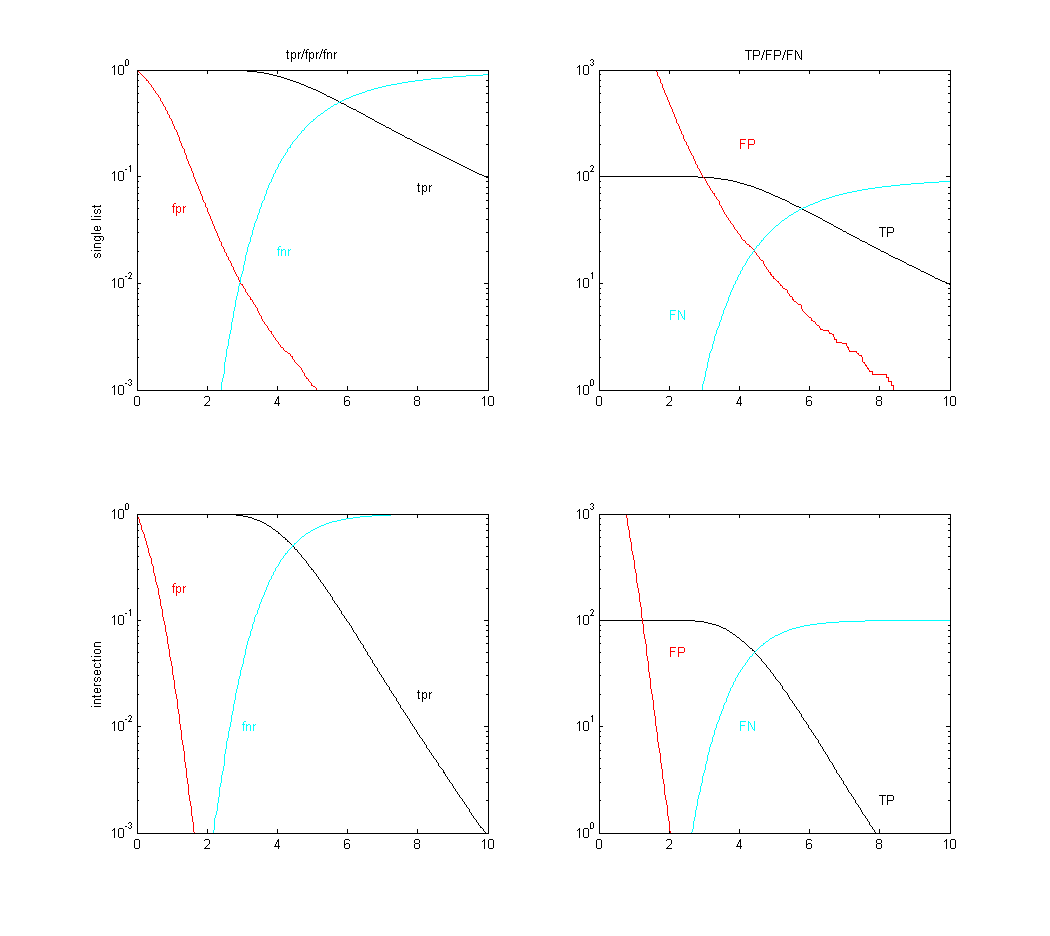

Figure 1: Rates (left column) and expected numbers (right column) for the individual lists (top row) and the intersection (bottom row) in a simulated set of three experiments; black=true positives, red=false positives and cyan=false negatives; parameter values given in the text, see also source Matlab code in SuppMat.

Let’s imagine a stringent criterion in which we expect each list to have no more than 10 false positives, so the p-value = fpr=0.001; for the (generous) numbers given above, this would set (see Figure 1) tpr=0.51, so we’d get 51 true positives and 10 false positives on each list. The intersection would only contain 13 correct genes and no false positive. This excessive stringency of the combined test is one basis for the small intersection problem. The situation becomes *substantially worse* if the statistical test is less powerful—e.g. if there were only two replicates or the fold-change were twofold rather than fourfold.

The process of intersecting lists made from an appropriate p_th_ is equivalent to unwisely choosing a p_th_ too stringent for the power of the test at hand. If a good intersection was desired, the optimal p_th_ for the intersection creates individual lists which are way too lax. Given the numbers above, a p-value of 0.001 for the intersection gives us 100 true positives and 10 false positives at the intersection, but each individual list has almost 1000 false positives (p=0.1, t-value 1.64). Therefore in order to get a good intersection the individual lists have to be recomputed.

## What causes poor accord between studies?

The statistical analysis of differentially expressed transcripts starts with a matrix of expression values. Obtaining this matrix is a long process, and all stages in this process, from choice of the base platform (e.g. Affymetrix vs. spot cDNA), experiment design, experimental conditions, technical expertise, labeling protocol, fluorescence imaging, spot quantitation and further data processing to the final gene expression values potentially contribute to differences in expression values. Once the matrix of expression values is obtained, then which approaches are used to assess differences in gene expression will also strongly affect the final “list of genes” that is presented.

A large number of recent studies have been addressing the agreement between platforms in the literature. Their results are often conflicting and do not support drawing definitive conclusions at this stage. This may be because the different causes of variation are overall not consistently controlled through these studies.

The spot intensity (the raw data) is quite affected by the platform used (oligonucleotides, cDNA). There are profound physical chemistry differences between them, in terms of length of binding and labeling methodology fundamentally; in addition, one should not consider cDNA “spot” arrays to be *one* platform but several, depending, e.g., on the provenance of the cDNA in question: whether curated sequence-certified standard libraries, or homebrewed differential libraries created for the tissue or species of interest, which may have substantial number of fragments or other artifacts frequently found in differential (subtractive) libraries. For instance, in the three studies we address in this paper, there is an Affymetrix study and two cDNA studies, yet the concordance is poorest amongst the two cDNA studies. The equipment and protocols for hybridazation, washing, scanning and image analysis are also frequent sources of variation between replications of experiments when carried out in a single platform.

^1^ and ^2^ based the comparison in the agreement of the lists generated by different statistical criteria and correlation between signal instead of M-values. ^3^ found poor correlation (<0.33) between M-value obtained by the cDNA and Affymetrix HU68chips in samples of cancer cell lines but since studies were carried out independently in two different laboratories, variations that may have arisen from independent cell culturing, RNA isolation and purification were not controlled.

^4^ and ^5^ found very good overall agreement among platforms. ^4^ compare two different collections of 70-mers spotted cDNA and Affymetrix HGU95av2 chips finding cross platform correlations of M-values of 0.8, rising to 0.89 when slow signals were eliminated. In ^5^ a broad evaluation of six platforms was presented. Pairwise comparison of the average M-values among replicates between long oligos techniques lead to correlations bigger than 0.7 and correlation between cDNA and Affymetrix were 0.52 and 0.58, comparables to those obtained by ^6^. Although ^6^ and ^5^ used the same statistical approach for each study and found moderately good correlation between M-values, they found poor agreement in the list of genes considered to be differentially expressed. Interestingly, in the first study PCA analysis suggested that the platform is the main source of variability while in the second, factorial analysis shows that 47.6% of the variance in the data is due to sample variability while 28.4% of the variance was explained by platform variability.

Agreement between platforms can be affected by the identification of common genes as ^3^ and ^7^ suggested in their studies. ^8^ show that sequence-matched probes increase cross-platform correlation between M-values. Although in a recent study ^9^ conclude that verification of sequence identity appears to play only a small role in the improvement of the result, their study was limited to the analysis of baseline quantitation of biological replicates and does not compare the arrays ability to detect changes.

Expression measures of Affymetrix genechips are sensitive to the algorithm used for the calculation and the use of different algorithms leads to different expression (Figure 1). ^8^ show that crossplatform correlation of M- values varies from 0.6 to 0.7 when RMA, MAS5 and dChip algorithms are used to compute expression values.


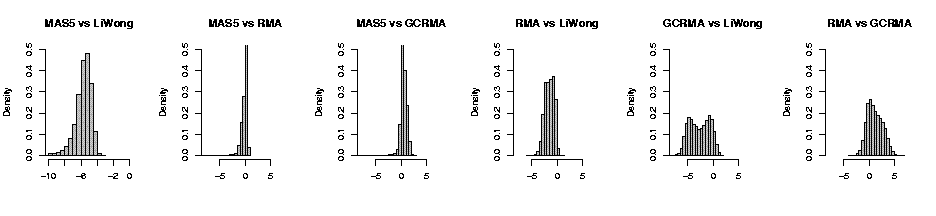
Figure 2. Histogram of the difference between expression measure values using different algorithms for Sato data, taken on Affymetrix Genechips HU133A.

## Description of the studies.

The Bhattacharya study has 6 chips. Different HESC lines were hybridized to the red channel (Cy5) of the arrays; 5 of them were lines BG01, BG02, GE01, GE09, TE06, and the sixth sample was a pool of GE01, GE07, and GE09. The control sample, hybridized to the green channel was “total human universal RNA (huURNA) isolated from a collection of adult human tissues to represent a broad range of expressed genes from both male and female donors (BD Biosciences, Palo Alto, CA)”. No replicates were performed for individual lines. The Sperger study used a similar design, hybridizing lines H1, H7, H13, H14 and two samples of H9. The control samples were “a common reference pool of mRNA”. The Sato study had 6 Affymetryx HGU133A chips, 3 replicates of H1 cells (in Matrigel/Conditioned Medium) and 3 replicates of “nonlineage-directed differentiation” (Matrigel/non-CM).

Table 1 summarizes the architecture of chips and the number of spot/genes involved in the three studies. Flagged spots were declared by the authors when deemed of insufficient quality. The image analysis algorithms for cDNA (GenePix 3.0 software (Axon Instruments, Union City, CA)). Spots with low quality were flagged too and genes with 3 or more low quality spots across chip were excluded from the analysis.

|  | Bhattacharya | Sperger | Sato |
| --- | --- | --- | --- |
|  | cDNA - | cDNA – 12x4 | Affy – HGU133a |
| Chip design | 8x4,23x23 | 12x4, 30x30 | 650x650 |
| Scanner | GenePix 4000B | GenePix 4000 | GeneArray |
| Image Analysis Soft | GenePix 3.0 | Gene Pix 3.0 | MAS Suite |
| Spot/probes (per chip) | 16 928 | 43200 / 43008 | 22 283 |
| Unigene | 12041 | 25 400 | 12 441 |
| Empty spots per chip | 238 | 531 |  |
| Flag spots | -9766 | -51 427 |  |
| Low quality spots | 125 | 5500 |  |
| Low quality genes | 736 | 724 |  |
| # of genes | 15 954 (94%) | 35 424 (82%) |  |

Table 1.Summary of Chip Designs and quantification of quality spots

## Results of Integrated Correlation Analysis

The integrated correlation coefficients between studies were extremely small: 0.058 between Bhattacharya-Sperger, 0.129 between Bhattacharya-Sato and 0.071 between Sperger-Sato, with tiny confidence intervals (see top of Figure 3). For each pair of studies, the two-dimensional density of the pairwise correlations is shown in Figure 3, which suggests that we can find many “negatively coherent” pairs of genes, positive correlated in one study and negatively correlated in the other, and in any such pair, one must be inconsistent. Inspection of correlation between M-values also indicates poor general agreement between studies: 0.32, 0.32, and 0.28 for Bhattacharya-Sperger, Bhattacharya-Sato, Sperger-Sato respectively. Those values are relatively similar to those reported in ^3^ nevertheless they can leads to genes with opposite results in the analysis.

|  | ρ=0.058, ICρ=[0.031,0.059] |  | ρ=0.13, ICρ=[0.054,0.131] |  | ρ=0.071, ICρ=[0.012,0.0710] |
| --- | --- | --- | --- | --- | --- |
| Sperger | 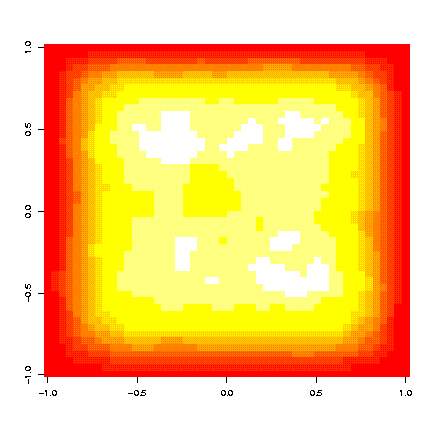 | Sato | 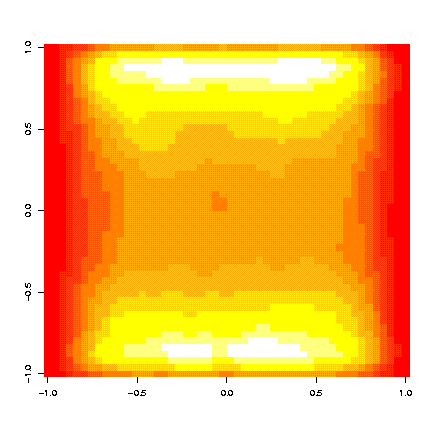 | Sato | 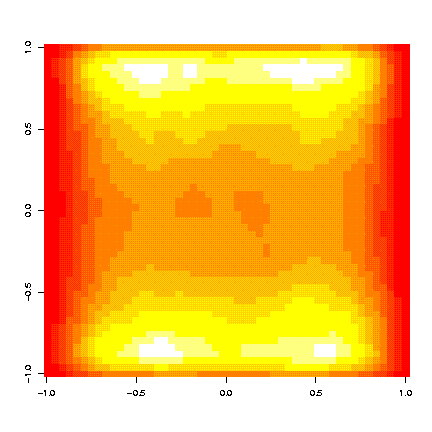 |
|  | Bhattacharya |  | Bhattacharya |  | Sperger |

Figure 3. Density of pairwise correlation between studies. On the top ρ is the integrated correlation between the studies and the confidence interval obtained trough bootstrapping. White is higher density

The histograms of the coherence scores between study pairs are shown in Figure 4, and paint a much more hopeful picture. We see the existence of a group of genes with high reproducibility scores in all study pairs. The histogram of the average pairwise reproducibility (Figure 4 b) shows a bimodal distribution, with an apparently clear-cut distinction between two groups of genes, one of them having positive reproducibility scores (“coherent”) and the other one close to zero (“erratics”) or negative (“incoherents”). So the general poor agreement observed between the studies is a result of averaging over a set of genes with both positive and negative coherences. We should note that there is a slight difference in pattern between the studies;

In Figure 5 we show the bivariate density of the coherence Score between pairs of studies (Univariate densities are the histograms of the Coherence score shown in Figure 4 a). We observed that despite variations, there is a group of genes where scores between Bhattacharya-Sperger are similar to the score of Bhattacharya-Sato, those that have higher values in both are part of the coherent set.

| Bhattacharya-Sperger, | Bhattacharya-Sato | Sperger-Sato |
| --- | --- | --- |
| 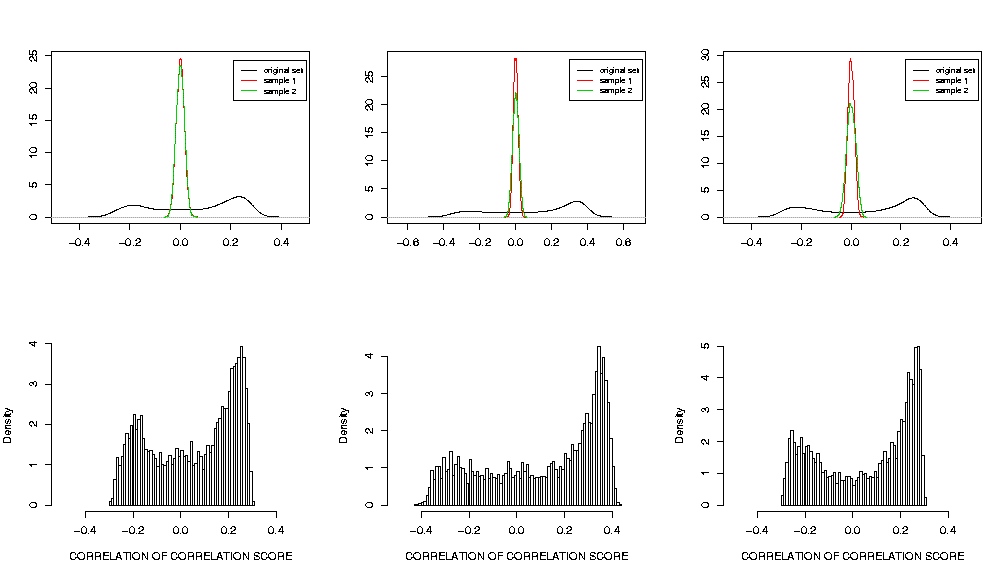 (a) | | |


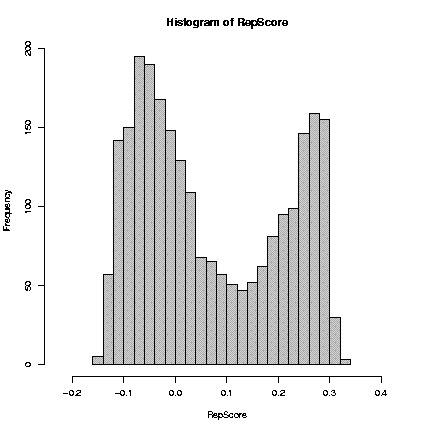
(b)

Figure 4. (a) Gene-Coherence score between the studies. In the first row, the density of the observed data and in green and red the density obtained from two random permutations of the columns of the expression matrix. The second row, contains the histograms. (b) Averaged score over all three comparisons.

| 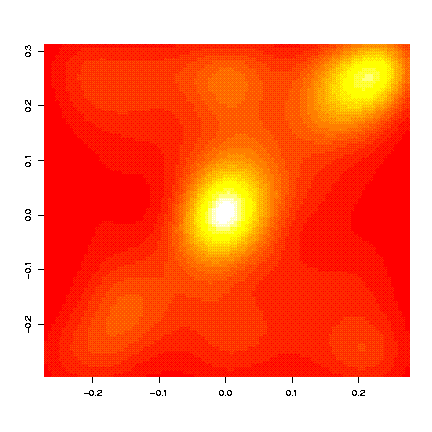 | 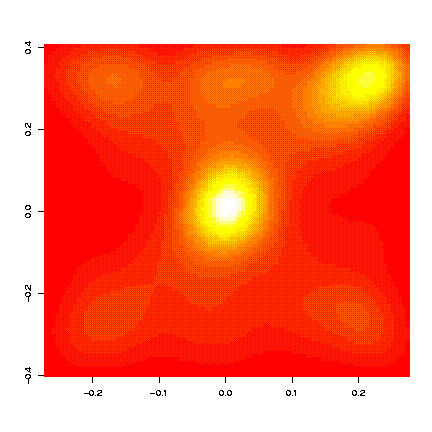 | 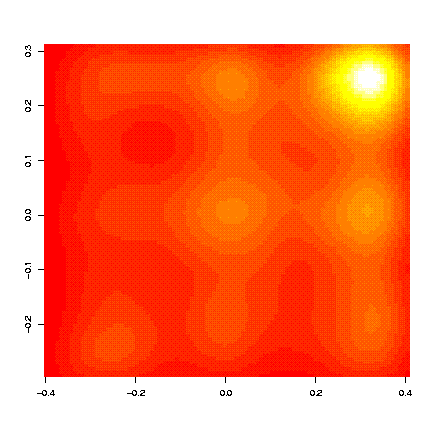 |
| --- | --- | --- |
| Bhattacharya-Sperger vs Bhattacharya-Sato | Bhattacharya-Sperger vs  Sperger-Sato | Sperger-Sato vs  Bhattacharya-Sato |
| Corr = 0.3 | Corr = 0.2 | Corr = 0.16 |

Figure 5. Bivariate densities of the Coherence Score.

## Selection of the set of consistent genes.

Eliminating erratic genes improves enormously the general agreement between the studies. The improvement in the integrated correlation and the correlation between M-values is illustrated in Figure 6. We decided to keep for further analysis the 739 genes in the top 30% of the gene-coherence distribution. The integrated correlation between studies, by definition, was much improved: Bhattacharya-Sperger:0.78, Bhattacharya-Sato:0.84 and Sperger-Sato:0.83. This improvement can be easily visualized if you compare the pairwise correlation densities shown in Figure 3 with the one in Figure 7, after the erratic genes are discarded. Not so obviously, the correlation between the M-values between studies also markedly improved to 0.76, 0.68, and 0.66 respectively. Correlations of the moderated t-statistics for the set of coherent genes are 0.76, 0.68, 0.68 respectively.


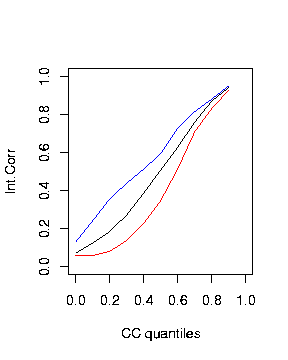

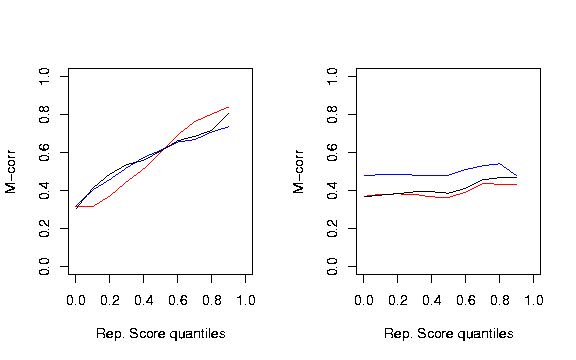


Figure 6. When “erratic” genes are discarded, there is a marked improvement in the agreement between studies. a) Integrated Correlation calculated using the genes in the top percentiles of Coherence Score. b) Correlation of M-values vs percentiles of RS. Red: Bhattacharya-Sperger, blue: Bhattacharya-Sato, black: Sperger-Sato.

| Sperger | 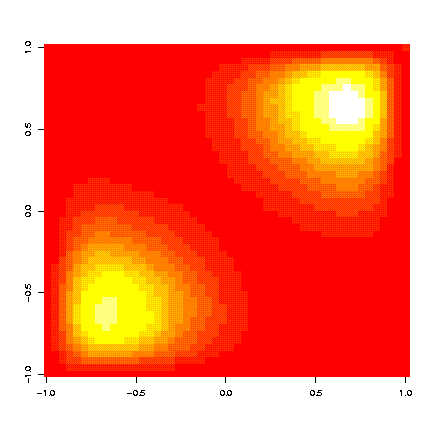 | Sato | 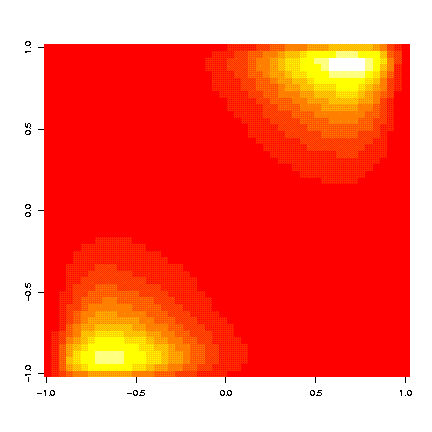 | Sato | 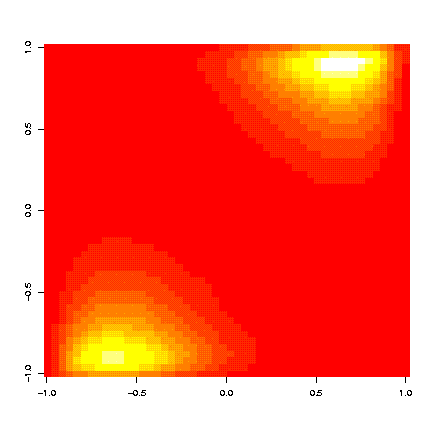 |
| --- | --- | --- | --- | --- | --- |
|  | Bhattacharya |  | Bhattacharya |  | Sperger |

Figure 7. Bivariate density of pairwise correlation between studies for the set of coherent genes

Table 2 shows how the correlation of the moderated t-statistics improve when the set is reduce to the most coherent genes.

Table 3 shows the number of gene in the intersection (up-regulated + down-regulated together) varies when the cutoff is p-values only or pvalues and lods positive for diferent p-values cutoff (p=0.01, p=0.1). Keep in mind that a p-value of 0.01 guarantees that the error for the pairwise intersection is 10-4 and 10-6 for the intersection of the three studies.

|  | All | 50% | 60% | 70% | 75% |
| --- | --- | --- | --- | --- | --- |
| Bhattacharya-Sperger | 0.31 | 0.65 | 0.71 | 0.76 | 0.77 |
| Bhattacharya-Sato | 0.30 | 0.64 | 0.66 | 0.68 | 0.71 |
| Sperger-Sato | 0.29 | 0.61 | 0.64 | 0.68 | 0.70 |

Table 2. Correlation between the moderated t-statistic for different set of genes. First column is for all the genes in the universe, 2^nd^ – 5^th^ are for the set of coherent genes defined as genes in the top 50^th^ -75^th^ percentile of the coherent-score distribution respectively

|  | P<0.01 | | | | | (P<0.01) (lods>0) | | | | |
| --- | --- | --- | --- | --- | --- | --- | --- | --- | --- | --- |
|  | All | 50% | 60% | 70% | 75% | All | 50% | 60% | 70% | 75% |
| Bhattacharya-Sperger | 810 | 474 | 485 | 472 | 431 | 273 | 430 | 420 | 398 | 369 |
| Bhattacharya-Sato | 428 | 294 | 292 | 282 | 268 | 172 | 278 | 270 | 261 | 238 |
| Sperger-Sato | 502 | 338 | 333 | 319 | 299 | 231 | 323 | 311 | 288 | 269 |
| all | 285 | 214 | 231 | 244 | 241 | 79 | 196 | 203 | 206 | 199 |

|  | P<0.1 | | | | | (P<0.1) (lods>0) | | | | |
| --- | --- | --- | --- | --- | --- | --- | --- | --- | --- | --- |
|  | All | 50% | 60% | 70% | 75% | All | 50% | 60% | 70% | 75% |
| Bhattacharya-Sperger | 1744 | 813 | 758 | 557 | 676 | 273 | 431 | 420 | 398 | 359 |
| Bhattacharya-Sato | 1174 | 671 | 630 | 660 | 499 | 172 | 283 | 270 | 261 | 238 |
| Sperger-Sato | 1261 | 677 | 637 | 661 | 611 | 231 | 329 | 311 | 299 | 269 |
| all | 1042 | 553 | 553 | 627 | 488 | 79 | 199 | 203 | 206 | 199 |

Table 3. Number of genes in the intersection by criteria used to select the significant ones. Rows are pairwise intersection and all is the intersection for the tree studies. Columns indicates First column is for all the genes in the universe, 2^nd^ – 5^th^ are for the set of coherent genes defined as genes in the top 50^th^ -75^th^ percentile of the coherent-score distribution respectively.

## What was discarded: genes with negative coherence score.

The histograms of reproducibility scores (Figure 3 and Figure 4) show that there are genes which have quite an inconsistent pattern across studies; furthermore, there seems to be some sort of clear distinction between these and covariant genes since the histograms are bimodal. Such behavior was not observed in ^10^, who reported an *unimodal* histogram of integrated correlations, with a very moderate number of genes having negative reproducibility scores of small magnitude; the authors of that study attributed the few negative cases to annotation errors. But in our comparison between studies, quite unexpectedly, a fairly large number of genes appear to behave opposite to one another in different studies while a similarly large number behaves consistently in all three studies.

There are many plausible biological reasons why genes could behave inconsistently, and also many artifactual ones. Genes involved in sensing or adapting to the environment may display inconsistent behavior because they pick up small differences in protocol; the maintenance system for HESCs still has many poorly understood variables, and whether cells are kept using feeder cells or CM may have profound impact. Then we should note that the cell lines employed in the studies *are actually all different*, so genes distinguishing these lines will naturally behave inconsistently. And, finally and perhaps the most plausible factor, not only are the cell lineages different, but *the reference states against which the three studies compare stem cells are all different.* Almost by definition of pluripotency, there are many more differentiated states than pluripotent states, and hence comparing against different differentiated states picks up the differences among them. Artifactual reasons may include annotation errors but also platform differences, which is suggested by the fact that the individual histograms of reproducibility scores show a smaller range and more marked peaks in the comparisons between spotted array and Affymetrix studies than the comparison between both spotted arrays, which shows a 30% wider range on both sides and a broader, less marked lower peak.

We then identified genes that have the most negative values of Coherence Score and analyzed them. These are genes that are statistically significant in all three studies but are up-regulated in one while down-regulated in others. The list is presented in Supporting Information – Table S8 and Table S9.

| Studies | FCH>2 | FCH>1.5 |
| --- | --- | --- |
| Bhattacharya - Sperger | 29 | 113 |
| Bhattacharya – Sato | 46 | 85 |
| Sperger-Sato | 35 | 89 |

# Methods in detail

Language and packages:

The statistical analysis was carried out in the R language version 2.0 (www.r-project.org), and packages were from the *Bioconductor* project (www.bioconductor.org). Gene Ontology analysis was carried out using EASE 2.0 software ^11^.

Raw Data:

Data from the Bhattacharya and Sato studies were obtained directly from the authors. The Sperger data were obtained through the Stanford Microarray Database (SMD, http://genome-www5.stanford.edu). cDNA array data were output files from GenePix 3.0. Affymetrix raw data files were .CEL files.

The Bhattacharya study has 6 chips. Different HESC lines were hybridized to the red channel (Cy5) of the arrays; 5 of them were lines BG01, BG02, GE01, GE09, TE06, and the sixth sample was a pool of GE01, GE07, and GE09. The control sample, hybridized to the green channel was “total human universal RNA (huURNA) isolated from a collection of adult human tissues to represent a broad range of expressed genes from both male and female donors (BD Biosciences, Palo Alto, CA)”. No replicates were performed for individual lines. The Sperger study used a similar design, hybridizing lines H1, H7, H13, H14 (all unreplicated), and two samples of H9. The control samples were “a common reference pool of mRNA”. The Sato study had 6 Affymetryx HGU133A chips, 3 replicates of H1 cells (in Matrigel/Conditioned Medium) and 3 replicates of “nonlineage-directed differentiation” (Matrigel/non-CM). Please note that *both* lines and controls were different across studies, as well as maintenance conditions (feeder cells vs. Matrigel/CM).

We used the same image analysis criteria to exclude low quality spots for cDNA arrays. Both channel with Signal >150 and Signal over Background bigger than 1.5. Clones with 3 or more low-quality spots across the set of arrays were excluded from the analysis.

Expression Measures:

cDNA: the *marray* package from the Bioconductor suite was used. In cDNA arrays, normalization was executed in two steps, first within-print-tip-group location-dependent intensity normalization followed by within-print-tip group scale normalization using median absolute deviation. Additionally, a 2D special normalization ^12^ was needed for one chip of the Bhattacharya study (5^th^ chip, sample PES.gpr). As we intended to work here with the intensity of each channel separately for the integrated correlation analysis, we needed to assure that the intensities have the same empirical distribution across array and across channels, so single-channel normalization of two-color cDNA was done as proposed by ^13^, using quantile normalization.

Affymetrix: the GCRMA algorithm was used to summarize Affymetrix data as proposed in ^14^. This algorithm improves the widely used RMA ^15^ by including an extra step to adjust for non-specific binding, and computing the sequence-specific affinities between probes as described ^16^. This algorithm outperforms both RMA and MAS5 (among others), leading to more precise estimators, especially for the low signal intensities. A complete comparison of the performance of GCRMA with other common algorithms for Affymetrix chips can be found in http://affycomp.biostat.jhsph.edu.

Within-platform variability

In order to assess the quality of the data replications the within platform variations were analyzed. For each study, the correlation matrix for ES, control and M-values (log_2_-fold changes) samples was computed, using all the unflagged spots. Mean correlation and generalized variance were reported as measurement of reproducibility within platform. Determinant of correlation matrix represent a measure of generalized variance of the standardized variables; generalized variance is near 0 when two or more variables are correlated and near 1 when the correlation is very low. Similar numbers were obtained using the set of common genes.

|  | Bhattacharya | | | Sperger | | | Sato | | |
| --- | --- | --- | --- | --- | --- | --- | --- | --- | --- |
|  | ES | Dif | M | ES | Dif | M | ES | Dif | M |
| Generalized Var. | 3.96x10^-3^ | 1.6x10^-3^ | 0.0127 | 10^-3^ | 1.1x10^-3^ | 0.036 | 4.2x10^-4^ | 1.65x10^-4^ | 0 |
| Mean Correlation | 0.803 | 0.841 | 0.68 | 0.891 | 0.893 | 0.6 | 0.990 | 0.978 | 0.85 |

Table 4. Statistics for the correlation matrix among replicates. Using all genes of sufficient quality for each studies

The within-study reproducibility is overall fairly good in all the studies, even noting that Bhattacharya ’s and Sperger’s design contain different lines of HESC rather than true replicates of a single line. The 6 green channels of both cDNA studies have better reproducibility than the ES samples since they are in both cases technical replicates, while the Sato study has the best sample reproducibility since they are replicates of the same line.

Annotations

For both Bhattacharya and Sperger studies, annotations were obtained from SOURCE from the Stanford microarray data homepage ([http://source.standford.edu](http://source.standford.edu/)). For Affymetrix data, annotations packages from Bioconductor were used. The IMAGE clone IDs and the Affymetrix probes were matched using Unigene Cluster Annotation. Genes with no Unigene number were eliminated from the study. Spots or probesets with duplicated Unigene identifiers were averaged together.

Common Genes

Annotations were obtained with the raw data from each study. Genes without Unigene identifier were eliminated and duplicated probes/spots were averaged together. After this process there are 7373 genes common to all 3 studies as shown Figure 8a. We filtered for evidence of variation across samples, reducing our set of interesting genes to those showed in Figure 8b. For the cDNA arrays, we select genes where the M-values was bigger than 0.3 in at least 4 arrays and in Affymetrix experiment we keep genes whose expression profile had range bigger than 0.5

(a)(b)

Figure 8.The universe of our study. a) Venn diagram of all good quality genes with assigned Unigene names. b) The genes of (a) after variation filter.

Coherent genes: the integrated correlation approach.

Integrated correlation analysis was introduced in ^10^ to validate the agreement across studies and to select the genes that exhibit a coherent behavior across the studies. The idea is that while studying the same system, coregulated genes should exhibit correlated expression profiles that should be maintained across studies. To quantify the reproducibility of the results across the studies the proposition is to examine all pairwise correlation of gene expression across the studies.

For each study s, let us define x_g_ the expression profile for a gene g, and$\rho_{p}^{s}=\text{corr}(x_{g_{1}},x_{g_{2}})$ the correlation for the pair of genes p=(g_1_,g_2_) in the study. Based on $\rho_{p}^{s}$ we can asset both overall coherence between studies and gene-specific coherence. The integrated correlation, defined as: $I(s,s')=\text{corr}(\rho_{p}^{s},\rho_{p}^{s'})$ quantified the coherence between studies. If this expression is calculated considering only the pairs containing a gene g, then we have a measure of the gene-specific coherence between two studies, that is $C^{s,s'}(g)=\text{corr}(\rho_{p}^{s},\rho_{p}^{s'})$, where p=(g,j). When more that two studies are involved, the average over all s and s’ is used as a Coherence Score for a gene g, .

To account for the existence of missing values in expression profiles, pairwise correlations (between genes) were calculated using all complete pair of observations. Both Pearson and Spearman correlations were tested and gave very similar results; the results using Pearson correlations are reported here. Confidence intervals for Integrated correlation score were obtained by bootstrapping.

Coherence and consistency.

In ^10^, the coherence score defined above is called reproducibility score and coherent genes (genes with high values of the score) are called “consistent”. However we once again stress that this score bears no direct logical relationship to the notion of reproducibility or consistency in the sense of consistent up- or down- regulation in both studies. A simple counterexample makes the point: create a fake Study 3 which is Study 2 with the values of the condition and control swapped; then by the above definitions, all genes are perfectly coherent for studies 2 and 3, having coherence (reproducibility) scores equal to 1, the maximum possible; yet each gene which is up-regulated in study 2 is down-regulated by the same amount in the (fictitious) study 3, so all genes are inconsistent. A relationship between the coherence scores and consistent behavior is predicated on the counter-reciprocal: if a pair of genes is incoherent then both genes cannot be consistent, and hence if a gene has a negative coherence (reproducibility) score it is “likely” (though by no means sure) to be inconsistent by being the “odd one out”.

Differentially Expression Criteria

Statistical analysis to determine which genes are differentially expressed was carried out using the package Limma from the Bioconductor project. For assessing differential expression the moderated t-statistics was used as proposed by ^17^ in all the 3 studies. To do so, Limma uses an empirical Bayes method to moderate the standard errors of the estimated log_2_-fold changes. This results in more stable inference and improved power, especially for experiments with small number of arrays ^17^. The p-values of the moderated t-test were adjusted for multiple hypothesis testing, controlling the false discovery rate (fdr) as proposed ^18^.

We use a strict cut off criterion for selectivity of the genes based on both the p-values and lods ratio, as proposed in ^19^. The lods (or B-statistic) is the log of the odds that the gene is differentially expressed. Odds ratios indicate if it is more likely that a gene is differentially expressed than not. Suppose for example that lods=1.5., the odds of differential expression is exp(1.5)=4.48, i.e. the about 4½. The probability than the gene is differentially expressed is 4.48/(1+4.48)=0.82, i.e., about 82%. A lods of zero correspond to 50-50 chance that the gene is differentially expressed. The B-statistic is automatically adjusted for multiple hypotheses assuming that approximately 10% of the genes out of he 723 are expected to be differentially expressed.

The moderated t-statistic and lods usually provide a very similar ranking of the genes, but depending of the p-value cutoff, the criteria of positive lods is usually more restrictive. For the set of coherent genes we selected here, the cutoff of p=0.01 and positive lods leads to 206 (111 up, 92 down) genes while 244 (139 up 105 down) is obtained considered only the p-value cut-off. In Supporting Information Document S1 the reader can find how the number of selected genes depends on the criteria for different set of coherent genes.

Real-time RT-PCR verification of gene expression level in HESCs:

Relative expression levels of the 106 (95%) of 111 genes in the intersection were analyzed in H1 HESCs maintained in CM or differentiated by withdrawal of CM for 30 days by real-time RT-PCR. Raw data were normalized to Ubiquitin-C expression and relative expression levels were determined using PCR efficiency-adjusted ratios ^20^.

H1 HESCs were grown in feeder free conditions in the presence of conditioned medium from MEFs (CM)^21^. To induce differentiation, CM was withdrawn and replaced with non-conditioned growth medium for 30 days. RNA was extracted using Trizol Reagent (Invitrogen) following the manufacturers instructions. RNA was DNAse treated with the DNAse-free kit from Ambion according to instructions. RNA was quantitated by fluorescence using RiboGreen (Molecular Probes). RT reactions were performed using the SuperScript III First-Strand Synthesis System (Invitrogen) according to instructions, with the following modifications. Anchored OligodT (Integrated DNA Technologies) oligos were used to prime first strand synthesis and DTT was omitted from the RT reaction ^22^. 50ng of cDNA was used in real-time PCR reactions using the SYBR Green Master Mix (Applied Biosystems) on an ABI Prism 7900HT Sequence Detection System. Primers were designed according to manufacturer’s guidelines (Applied Biosystems) and screened for amplification of a single product by melting curve analysis (see Supporting Information TableS5 for Primer sequences) . We were able to verify good primer sets for 106 of 111 genes. Ubiquitin-C (UBC) was used as an internal control for normalization as described ^20^ except that corrections were included for PCR amplification efficiencies and calculated as described ^23^. Our testing using previously published methodology ^24^ has found that, under our culture conditions and treatments, GAPDH is one of the least stably expressed housekeeping genes, with Ubiquitin-C (UBC), beta-2-microglobulin, and HPRT among the most stable. Reactions for each primer set were run in triplicate. Student’ t-test was used to access significance of differences in delta Ct values between differentiated and undifferentiated samples.

# References

1. Li, J., Pankratz, M. & Johnson, J. Differential gene expression patterns revealed by oligonucleotide versus long cDNA arrays. TOXICOLOGICAL SCIENCES 69, 383-390 (2002).

2. Kothapalli, R., Yoder, S., Mane, S. & Loughran, T. Microarray results: how accurate are they? BMC BIOINFORMATICS 3, 22 (2002).

3. Kuo, W., Jenssen, T., Butte, A., Ohno-Machado, L. & Kohane, I. Analysis of matched mRNA measurements from two different microarray technologies. BIOINFORMATICS 18, 405-412 (2002).

4. Barczak, A. et al. Spotted long oligonucleotide arrays for human gene expression analysis. GENOME RESEARCH 13, 1775-1785 (2003).

5. Yauk, C., Berndt, M., Williams, A. & Douglas, G. Comprehensive comparison of six microarray technologies. NUCLEIC ACIDS RESEARCH 32, e124 (2004).

6. Tan, P. et al. Evaluation of gene expression measurements from commercial microarray platforms. NUCLEIC ACIDS RESEARCH 31, 5676-5684 (2003).

7. Yuen, T., Wurmbach, E., Pfeffer, R., Ebersole, B. & Sealfon, S. Accuracy and calibration of commercial oligonucleotide and custom cDNA microarrays. NUCLEIC ACIDS RESEARCH 30, e48- (2002).

8. Mecham, B. et al. Sequence-matched probes produce increased cross-platform consistency and more reproducible biological results in microarray-based gene expression measurements. NUCLEIC ACIDS RESEARCH 32, e74- (2004).

9. Mah, N. et al. A comparison of oligonucleotide and cDNA-based microarray systems. PHYSIOLOGICAL GENOMICS 16, 361-370 (2004).

10. Parmigiani, G., Garrett-Mayer, E., Anbazhagan, R. & Gabrielson, E. A cross-study comparison of gene expression studies for the molecular classification of lung cancer. CLINICAL CANCER RESEARCH 10, 2922-2927 (2004).

11. Hosack, D., Dennis, G., Sherman, B., Lane, H. & Lempicki, R. Identifying biological themes within lists of genes with EASE. GENOME BIOLOGY 4, 1465-6909 (2003).

12. Yang, Y. H. et al. Normalization for cDNA microarray data: a robust composite method addressing single and multiple slide systematic variation. Nucl. Acids Res. 30, e15- (2002).

13. Yang, Y. H. & Thorne, N. in Science and Statistics: A Festschrift for Terry Speed

(ed. Goldstein, D. R.) 403-418 (2003).

14. Wu, Z., Irizarry, R., Gentleman, R., Martinez Murillo, F. & Spencer, F. A Model Based Background Adjustement for Oligonucleotide Expression Arrays. Journal of American Statistical Association 99, 909-917 (2004).

15. Irizarry, R. A. et al. Summaries of Affymetrix GeneChip probe level data. Nucl. Acids Res. 31, e15- (2003).

16. Naef, F. & Magnasco, M. Solving the riddle of the bright mismatches: Labeling and effective binding in oligonucleotide arrays. PHYSICAL REVIEW E 68, - (2003).

17. Smyth, G. K. Linear models and empirical Bayes methods for assessing differential expression in microarray experiments. Statistical Applications in Genetics and Molecular Biology

3, Article 3 (2004).

18. Dudoit, S., Shaffer, J. & Boldrick, J. Multiple hypothesis testing in microarray experiments. Statistical Science 18, 71-103 (2003).

19. Lonnstedt, I. & Speed, T. Replicated microarray data. STATISTICA SINICA 12, 31-46 (2002).

20. Pfaffl, M. W. A new mathematical model for relative quantification in real-time RT-PCR. Nucleic Acids Res 29, e45 (2001).

21. Xu, C. et al. Feeder-free growth of undifferentiated human embryonic stem cells. Nat Biotechnol 19, 971-4 (2001).

22. Lekanne Deprez, R. H., Fijnvandraat, A. C., Ruijter, J. M. & Moorman, A. F. Sensitivity and accuracy of quantitative real-time polymerase chain reaction using SYBR green I depends on cDNA synthesis conditions. Anal Biochem 307, 63-9 (2002).

23. Ramakers, C., Ruijter, J. M., Deprez, R. H. & Moorman, A. F. Assumption-free analysis of quantitative real-time polymerase chain reaction (PCR) data. Neurosci Lett 339, 62-6 (2003).

24. Vandesompele, J. et al. Accurate normalization of real-time quantitative RT-PCR data by geometric averaging of multiple internal control genes. Genome Biol 3, 34.31-34 (2002).

### Supporting Information for this Document

S8. List of differentiated genes with fold change bigger than 2 and incoherent behavior (per pair of study).

S9. List of differentiated genes with fold change bigger than 1.5 and incoherent behavior (per pair of study).
