# Supplementary material for: Comparing independent microarray studies: the case of human embryonic stem cells
Source: BMC Genomics. 2005 Jul 22;6:99. doi: 10.1186/1471-2164-6-99 (PMC1183205; doi:10.1186/1471-2164-6-99)
Supplement: Additional File 2 — Up-regulated genes in the intersection. List of up-regulated genes in the intersection of the 3 studies. In html format, including annotations and links. [file 1471-2164-6-99-S2.html]

Coherent and Significantly UP-regulated Genes across all studies


# Coherent and Significantly UP-regulated Genes across all studies

| Probe | Symbol | Description | Function | GenBank | LocusLink | UniGene | Gene Ontology | Pathway | Mmean | FCHmean | Bmean | ES-Bhatt | ES-Sperger | ES-Sato |
| --- | --- | --- | --- | --- | --- | --- | --- | --- | --- | --- | --- | --- | --- | --- |
| 208286\_x\_at | POU5F1 | POU domain, class 5, transcription factor 1 |  | NM\_002701 | 5460 | Hs.249184 |  |  | 4.00367 | 16.0408 | 9.98561 | 13.9555 | 12.6929 | 12.8027 |
| 219740\_at | FLJ12505 | hypothetical protein FLJ12505 |  | NM\_024749 | 79805 | Hs.96885 |  |  | 3.58994 | 12.0415 | 6.45477 | 11.3032 | 9.70735 | 8.99938 |
| 205100\_at | GFPT2 | glutamine-fructose-6-phosphate transaminase 2 |  | NM\_005110 | 9945 | Hs.30332 |  | Glutamate metabolism  Aminosugars metabolism | 2.64924 | 6.27335 | 9.69009 | 11.0672 | 10.9296 | 9.6311 |
| 205350\_at | CRABP1 | cellular retinoic acid binding protein 1 |  | NM\_004378 | 1381 | Hs.346950 |  |  | 2.61042 | 6.10683 | 8.20369 | 11.7037 | 12.8687 | 9.20497 |
| 206012\_at | EBAF | endometrial bleeding associated factor (left-right determination, factor A; transforming growth factor beta superfamily) |  | NM\_003240 | 7044 | Hs.25195 | transforming growth factor beta receptor binding  oocyte axis determination  transforming growth factor beta receptor signaling pathway  cell-cell signaling  cell growth  growth |  | 2.50776 | 5.68735 | 3.79629 | 9.57936 | 10.042 | 11.1942 |
| 201578\_at | PODXL | podocalyxin-like |  | NM\_005397 | 5420 | Hs.16426 | integral to plasma membrane |  | 2.5 | 5.65685 | 8.39565 | 12.5629 | 14.7365 | 12.6654 |
| 213283\_s\_at | SALL2 | sal-like 2 (Drosophila) |  | BG285616 | 6297 | Hs.416358 | transcription factor activity  regulation of transcription, DNA-dependent  nucleus |  | 2.47654 | 5.56561 | 9.27796 | 9.86598 | 12.8574 | 10.6691 |
| 201601\_x\_at | IFITM1 | interferon induced transmembrane protein 1 (9-27) |  | NM\_003641 | 8519 | Hs.458414 | receptor signaling protein activity  negative regulation of cell proliferation  cell surface receptor linked signal transduction  immune response  regulation of cell cycle  integral to membrane  plasma membrane |  | 2.37398 | 5.18368 | 9.38159 | 12.0349 | 14.0525 | 11.4376 |
| 202234\_s\_at | SLC16A1 | solute carrier family 16 (monocarboxylic acid transporters), member 1 |  | BF511091 | 6566 | Hs.75231 | mevalonate transporter activity  mevalonate transport  transport  integral to membrane  membrane fraction  monocarboxylate porter activity  symporter activity |  | 2.23393 | 4.70414 | 7.58832 | 13.1663 | 13.7318 | 10.3626 |
| 209848\_s\_at | SILV | silver homolog (mouse) |  | U01874 | 6490 | Hs.95972 | melanin biosynthesis from tyrosine  integral to membrane  extracellular space  plasma membrane |  | 2.21139 | 4.6312 | 6.33275 | 9.63811 | 12.6474 | 7.18162 |
| 205924\_at | RAB3B | RAB3B, member RAS oncogene family |  | BC005035 | 5865 | Hs.123072 | GTP binding  RAB small monomeric GTPase activity  protein transporter activity  small GTPase mediated signal transduction  intracellular protein transport |  | 2.13401 | 4.38935 | 7.9965 | 9.33258 | 10.3581 | 6.3124 |
| 219464\_at | CA14 | carbonic anhydrase XIV |  | NM\_012113 | 23632 | Hs.192491 | carbonate dehydratase activity  zinc ion binding  integral to membrane  one-carbon compound metabolism  lyase activity | Nitrogen metabolism | 2.0592 | 4.16756 | 7.74017 | 10.3198 | 10.7593 | 8.3369 |
| 201969\_at | NASP | nuclear autoantigenic sperm protein (histone-binding) |  | AW003362 | 4678 | Hs.446206 | DNA packaging  spermatogenesis  nucleus |  | 2.03412 | 4.09573 | 7.22105 | 11.782 | 11.1564 | 10.7773 |
| 208650\_s\_at | CD24 | CD24 antigen (small cell lung carcinoma cluster 4 antigen) |  | BG327863 | 934 | Hs.375108 | humoral immune response  plasma membrane |  | 2.01614 | 4.045 | 8.04776 | 12.2905 | 13.4553 | 12.993 |
| 204269\_at | PIM2 | pim-2 oncogene |  | NM\_006875 | 11040 | Hs.80205 | ATP binding  protein serine/threonine kinase activity  protein amino acid phosphorylation  transferase activity |  | 2.00829 | 4.02306 | 6.92992 | 12.0361 | 12.9758 | 9.66487 |
| 203764\_at | DLG7 | discs, large homolog 7 (Drosophila) |  | NM\_014750 | 9787 | Hs.77695 | molecular\_function unknown  biological\_process unknown  cell-cell signaling  cellular\_component unknown |  | 1.96214 | 3.8964 | 6.96943 | 11.6076 | 11.4063 | 11.2605 |
| 205961\_s\_at | PSIP1 | PC4 and SFRS1 interacting protein 1 |  | NM\_004682 | 11168 | Hs.351305 |  |  | 1.94184 | 3.84196 | 7.72109 | 10.6594 | 9.81281 | 10.784 |
| 206136\_at | FZD5 | frizzled homolog 5 (Drosophila) |  | NM\_003468 | 7855 | Hs.152251 | G-protein coupled receptor activity  establishment of tissue polarity  G-protein coupled receptor protein signaling pathway  development  integral to plasma membrane  Wnt receptor signaling pathway  non-G-protein coupled 7TM receptor activity |  | 1.92288 | 3.79179 | 4.02111 | 8.9457 | 9.41367 | 5.11048 |
| 205480\_s\_at | UGP2 | UDP-glucose pyrophosphorylase 2 |  | NM\_006759 | 7360 | Hs.417361 | UTP-glucose-1-phosphate uridylyltransferase activity  UDP-glucose metabolism  metabolism  kinase activity  transferase activity | Pentose and glucuronate interconversions  Galactose metabolism  Starch and sucrose metabolism  Nucleotide sugars metabolism | 1.91942 | 3.78271 | 6.28132 | 12.3252 | 11.8883 | 13.1313 |
| 201831\_s\_at | VDP | vesicle docking protein p115 |  | BE875592 | 8615 | Hs.325948 | protein transporter activity  vesicle docking during the process of exocytosis  intracellular protein transport  membrane  Golgi membrane |  | 1.82984 | 3.55497 | 4.15074 | 10.6516 | 10.54 | 10.8457 |
| 221591\_s\_at | FLJ10156 | hypothetical protein FLJ10156 |  | BC005004 | 54478 | Hs.404323 |  |  | 1.82237 | 3.53661 | 6.75124 | 9.60896 | 9.86916 | 9.43391 |
| 218517\_at | JADE1 | PHD protein Jade-1 |  | NM\_024900 | 79960 | Hs.12420 |  |  | 1.80839 | 3.50251 | 7.35418 | 11.2824 | 11.8007 | 8.79593 |
| 210963\_s\_at | GYG2 | glycogenin 2 |  | U94363 | 8908 | Hs.380757 | glycogenin glucosyltransferase activity  glycogen biosynthesis  soluble fraction  carbohydrate biosynthesis  transferase activity  transferase activity, transferring hexosyl groups |  | 1.78853 | 3.45463 | 6.17886 | 11.0325 | 11.3988 | 8.14659 |
| 206891\_at | ACTN3 | actinin, alpha 3 |  | NM\_001104 | 89 | Hs.445037 | structural constituent of muscle  actin binding  calcium ion binding  actin filament |  | 1.78336 | 3.44226 | 4.98063 | 9.15234 | 11.2702 | 6.70225 |
| 215028\_at | SEMA6A | sema domain, transmembrane domain (TM), and cytoplasmic domain, (semaphorin) 6A |  | AB002438 | 57556 | Hs.443012 | protein binding  axon guidance  cytoskeleton organization and biogenesis  cell surface receptor linked signal transduction  development  neurogenesis  apoptosis  integral to membrane  axon |  | 1.74147 | 3.34377 | 5.07253 | 10.217 | 9.34025 | 8.7091 |
| 202345\_s\_at | FABP5 | fatty acid binding protein 5 (psoriasis-associated) |  | NM\_001444 | 2171 | Hs.408061 | transporter activity  fatty acid binding  epidermal differentiation  transport  lipid metabolism  cytoplasm |  | 1.71899 | 3.29207 | 6.1885 | 12.4277 | 13.88 | 13.1939 |
| 203744\_at | HMGB3 | high-mobility group box 3 |  | NM\_005342 | 3149 | Hs.19114 | DNA binding  regulation of transcription, DNA-dependent  nucleus  chromatin |  | 1.6736 | 3.19009 | 7.23907 | 10.2727 | 11.7087 | 11.7683 |
| 204304\_s\_at | PROM1 | prominin 1 |  | NM\_006017 | 8842 | Hs.370052 | vision  integral to plasma membrane |  | 1.65859 | 3.15708 | 7.08007 | 9.72209 | 11.1314 | 10.5474 |
| 209433\_s\_at | PPAT | phosphoribosyl pyrophosphate amidotransferase |  | AI457120 | 5471 | Hs.311625 | amidophosphoribosyltransferase activity  purine nucleotide biosynthesis  purine base biosynthesis  nucleoside metabolism  metabolism  magnesium ion binding  glutamine metabolism  transferase activity, transferring glycosyl groups | Purine metabolism  Glutamate metabolism | 1.60526 | 3.0425 | 6.37596 | 10.8696 | 10.7554 | 9.14889 |
| 221763\_at | TRIP8 | thyroid hormone receptor interactor 8 |  | AI694023 | 221037 | Hs.442675 | regulation of transcription, DNA-dependent  intracellular  ligand-dependent thyroid hormone receptor interactor activity  thyroid hormone receptor binding |  | 1.6029 | 3.03754 | 6.31368 | 11.1984 | 10.6649 | 10.5624 |
| 203917\_at | CXADR | coxsackie virus and adenovirus receptor |  | NM\_001338 | 1525 | Hs.79187 |  |  | 1.58151 | 2.99284 | 5.64884 | 10.2142 | 11.3043 | 12.3638 |
| 220116\_at | KCNN2 | potassium intermediate/small conductance calcium-activated channel, subfamily N, member 2 |  | NM\_021614 | 3781 | Hs.98280 | ion channel activity  calmodulin binding  potassium ion transport  ion transport  integral to membrane  small conductance calcium-activated potassium channel activity |  | 1.56228 | 2.95321 | 3.29724 | 9.33798 | 10.0724 | 7.80195 |
| 209193\_at | PIM1 | pim-1 oncogene |  | M24779 | 5292 | Hs.81170 | ATP binding  protein serine/threonine kinase activity  cell growth and/or maintenance  protein amino acid phosphorylation  development  cytoplasm  nucleus  transferase activity |  | 1.53491 | 2.8977 | 3.76213 | 10.6237 | 11.0046 | 8.70628 |
| 219004\_s\_at | C21orf45 | chromosome 21 open reading frame 45 |  | NM\_018944 | 54069 | Hs.49932 | molecular\_function unknown  biological\_process unknown  cellular\_component unknown |  | 1.51756 | 2.86307 | 6.32901 | 10.9312 | 11.0101 | 9.1669 |
| 213721\_at | SOX2 | SRY (sex determining region Y)-box 2 |  | L07335 | 6657 | Hs.816 | transcription factor activity  establishment and/or maintenance of chromatin architecture  regulation of transcription, DNA-dependent  nucleus |  | 1.47485 | 2.77955 | 5.04765 | 11.8013 | 13.0813 | 6.23121 |
| 208810\_at | DNAJB6 | DnaJ (Hsp40) homolog, subfamily B, member 6 |  | AF080569 | 10049 | Hs.181195 | heat shock protein activity  biological\_process unknown  cellular\_component unknown |  | 1.41925 | 2.67447 | 4.61097 | 10.9669 | 12.3626 | 10.3963 |
| 204595\_s\_at | STC1 | stanniocalcin 1 |  | AI300520 | 6781 | Hs.25590 | hormone activity  response to nutrients  calcium ion homeostasis  cell surface receptor linked signal transduction  cell-cell signaling  extracellular |  | 1.4144 | 2.66549 | 2.93965 | 9.56129 | 9.85415 | 5.97747 |
| 204235\_s\_at | GULP1 | GULP, engulfment adaptor PTB domain containing 1 |  | AF200715 | 51454 | Hs.107056 | signal transducer activity  phagocytosis, engulfment  apoptosis |  | 1.41138 | 2.65992 | 4.24958 | 8.83448 | 11.681 | 9.28896 |
| 204832\_s\_at | BMPR1A | bone morphogenetic protein receptor, type IA |  | NM\_004329 | 657 | Hs.2534 | ATP binding  protein serine/threonine kinase activity  receptor activity  transforming growth factor beta receptor signaling pathway  protein amino acid phosphorylation  integral to membrane  transforming growth factor beta receptor activity  transferase activity |  | 1.39323 | 2.62666 | 4.5236 | 10.123 | 10.4829 | 9.79395 |
| 209722\_s\_at | SERPINB9 | serine (or cysteine) proteinase inhibitor, clade B (ovalbumin), member 9 |  | L40378 | 5272 | Hs.104879 | protein binding  serine-type endopeptidase inhibitor activity  cytosol |  | 1.38179 | 2.60591 | 3.71659 | 10.033 | 11.1833 | 8.41395 |
| 211020\_at | GCNT2 | glucosaminyl (N-acetyl) transferase 2, I-branching enzyme |  | L19659 | 2651 | Hs.934 | N-acetyllactosaminide beta-1,6-N-acetylglucosaminyltransferase activity  acetylglucosaminyltransferase activity  glycosaminoglycan biosynthesis  O-linked glycosylation  development  membrane  Golgi apparatus  integral to membrane  membrane fraction  transferase activity, transferring glycosyl groups | Blood group glycolipid biosynthesis-neolactoseries | 1.38151 | 2.60541 | 3.58192 | 8.5313 | 9.71348 | 5.80907 |
| 218885\_s\_at | GALNT12 | UDP-N-acetyl-alpha-D-galactosamine |  | NM\_024642 | 79695 | Hs.47099 | transferase activity |  | 1.37502 | 2.59371 | 4.89627 | 9.34559 | 10.4 | 8.59524 |
| 220028\_at | ACVR2B | activin A receptor, type IIB |  | NM\_001106 | 93 | Hs.23994 | ATP binding  protein binding  receptor activity  transmembrane receptor protein serine/threonine kinase signaling pathway  protein amino acid phosphorylation  integral to plasma membrane  transforming growth factor beta receptor activity  transferase activity |  | 1.37081 | 2.58616 | 3.51636 | 11.1975 | 11.3895 | 8.56135 |
| 208711\_s\_at | CCND1 | cyclin D1 (PRAD1 |  | BC000076 | 595 | Hs.371468 | G1/S transition of mitotic cell cycle  cell growth and/or maintenance  regulation of cell cycle  nucleus  cellular\_component unknown  cytokinesis | Cell cycle | 1.37079 | 2.58611 | 4.69409 | 11.6888 | 7.59103 | 8.86338 |
| 204559\_s\_at | LSM7 | LSM7 homolog, U6 small nuclear RNA associated (S. cerevisiae) |  | NM\_016199 | 51690 | Hs.512610 | pre-mRNA splicing factor activity  RNA binding  small nucleolar ribonucleoprotein complex  nucleus  U6 snRNA binding  nuclear mRNA splicing, via spliceosome |  | 1.30753 | 2.47518 | 5.01683 | 10.0156 | 11.7442 | 10.7689 |
| 217919\_s\_at | MRPL42 | mitochondrial ribosomal protein L42 |  | BE782148 | 28977 | Hs.331202 | structural constituent of ribosome  protein biosynthesis  mitochondrion  mitochondrial small ribosomal subunit |  | 1.29976 | 2.46189 | 3.71884 | 10.087 | 11.5158 | 11.3501 |
| 206055\_s\_at | SNRPA1 | small nuclear ribonucleoprotein polypeptide A' |  | NM\_003090 | 6627 | Hs.434901 | RNA binding  RNA splicing  snRNP U2 |  | 1.29926 | 2.46102 | 5.68961 | 11.582 | 13.2796 | 10.0474 |
| 205167\_s\_at | CDC25C | cell division cycle 25C |  | NM\_001790 | 995 | Hs.656 | protein-tyrosine-phosphatase activity  regulation of mitosis  regulation of CDK activity  start control point of mitotic cell cycle  protein amino acid dephosphorylation  nucleus  hydrolase activity  cytokinesis | Phosphatidylinositol signaling system  Cell cycle | 1.29605 | 2.45556 | 3.38601 | 9.17874 | 10.0726 | 7.04921 |
| 205895\_s\_at | NOLC1 | nucleolar and coiled-body phosphoprotein 1 |  | NM\_004741 | 9221 | Hs.75337 | GTP binding  ATP binding  rRNA processing  cell cycle  mitosis  nucleolus  cytoplasm |  | 1.27536 | 2.42059 | 2.89074 | 11.4568 | 10.4564 | 9.56213 |
| 209642\_at | BUB1 | BUB1 budding uninhibited by benzimidazoles 1 homolog (yeast) |  | AF043294 | 699 | Hs.287472 | ATP binding  protein serine/threonine kinase activity  mitotic spindle checkpoint  cell cycle  mitosis  protein amino acid phosphorylation  spindle pole body  nucleus  transferase activity  kinetochore | Starch and sucrose metabolism  Inositol phosphate metabolism  Sphingoglycolipid metabolism  Benzoate degradation via CoA ligation  Nicotinate and nicotinamide metabolism  Cell cycle | 1.26139 | 2.39726 | 3.80059 | 9.20591 | 11.7067 | 7.85922 |
| 218738\_s\_at | RNF138 | ring finger protein 138 |  | NM\_016271 | 51444 | Hs.180403 |  |  | 1.25079 | 2.37971 | 5.2997 | 9.62747 | 10.4421 | 11.5759 |
| 218781\_at | SMC6L1 | SMC6 structural maintenance of chromosomes 6-like 1 (yeast) |  | NM\_024624 | 79677 | Hs.424559 | ATP binding  chromosome segregation |  | 1.24384 | 2.36829 | 3.62334 | 10.2207 | 9.21581 | 8.8076 |
| 202330\_s\_at | UNG | uracil-DNA glycosylase |  | NM\_003362 | 7374 | Hs.78853 | uracil DNA N-glycosylase activity  base-excision repair  carbohydrate metabolism  mitochondrion  nucleus  hydrolase activity, acting on glycosyl bonds |  | 1.23699 | 2.35706 | 3.2107 | 9.98378 | 10.6047 | 10.4499 |
| 204766\_s\_at | NUDT1 | nudix (nucleoside diphosphate linked moiety X)-type motif 1 |  | NM\_002452 | 4521 | Hs.413078 | GTPase activity  response to oxidative stress  DNA repair  8-oxo-7,8-dihydroguanine triphosphatase activity  hydrolase activity |  | 1.22676 | 2.3404 | 4.26235 | 9.65397 | 12.3003 | 8.3917 |
| 218877\_s\_at | C6orf75 | chromosome 6 open reading frame 75 |  | NM\_021820 | 60487 | Hs.282575 |  |  | 1.21775 | 2.32583 | 5.71232 | 9.05781 | 10.606 | 9.17408 |
| 213213\_at | DATF1 | death associated transcription factor 1 |  | AL035669 | 11083 | Hs.438300 | DNA binding  transcription  regulation of transcription, DNA-dependent  apoptosis  nucleus |  | 1.20844 | 2.31088 | 4.6424 | 9.3304 | 10.5638 | 8.25762 |
| 201896\_s\_at | CKS1B | CDC28 protein kinase regulatory subunit 1B |  | BC001425 | 1163 | Hs.374378 | cyclin-dependent protein kinase activity  regulation of CDK activity  cytokinesis |  | 1.19585 | 2.2908 | 3.92594 | 10.8687 | 13.4517 | 10.3908 |
| 219433\_at | BCOR | BCL6 co-repressor |  | NM\_017745 | 54880 | Hs.186424 |  |  | 1.19545 | 2.29017 | 3.34357 | 9.17106 | 10.0839 | 7.25201 |
| 218283\_at | SS18L2 | synovial sarcoma translocation gene on chromosome 18-like 2 |  | NM\_016305 | 51188 | Hs.9774 |  |  | 1.1947 | 2.28897 | 5.62217 | 10.8996 | 10.7701 | 9.78467 |
| 203345\_s\_at | M96 | likely ortholog of mouse metal response element binding transcription factor 2 |  | AI566096 | 22823 | Hs.31016 | DNA binding  regulation of transcription, DNA-dependent |  | 1.18953 | 2.28079 | 5.74642 | 10.3016 | 10.4253 | 9.65946 |
| 218602\_s\_at | FAM29A | family with sequence similarity 29, member A |  | NM\_017645 | 54801 | Hs.54617 |  |  | 1.17955 | 2.26506 | 2.1953 | 9.45257 | 9.42787 | 7.54644 |
| 204510\_at | CDC7 | CDC7 cell division cycle 7 (S. cerevisiae) |  | NM\_003503 | 8317 | Hs.28853 | ATP binding  protein serine/threonine kinase activity  G1/S transition of mitotic cell cycle  negative regulation of cell proliferation  start control point of mitotic cell cycle  cell cycle  DNA replication initiation  protein amino acid phosphorylation  cytoplasm  nucleus  transferase activity  cytokinesis | Starch and sucrose metabolism  Inositol phosphate metabolism  Sphingoglycolipid metabolism  Benzoate degradation via CoA ligation  Nicotinate and nicotinamide metabolism  Cell cycle | 1.17072 | 2.25123 | 2.80817 | 9.24931 | 10.6044 | 8.51884 |
| 218878\_s\_at | SIRT1 | sirtuin (silent mating type information regulation 2 homolog) 1 (S. cerevisiae) |  | NM\_012238 | 23411 | Hs.31176 | DNA binding  chromatin silencing  myogenesis  regulation of transcription, DNA-dependent  apoptosis  nucleus  hydrolase activity  chromatin silencing complex |  | 1.16632 | 2.24438 | 3.5756 | 10.0848 | 9.76149 | 9.62374 |
| 202778\_s\_at | ZNF198 | zinc finger protein 198 |  | NM\_003453 | 7750 | Hs.315241 | zinc ion binding  regulation of transcription, DNA-dependent  biological\_process unknown  nucleus  cellular\_component unknown |  | 1.1662 | 2.2442 | 4.98973 | 9.34087 | 10.4454 | 7.07181 |
| 203832\_at | SNRPF | small nuclear ribonucleoprotein polypeptide F |  | NM\_003095 | 6636 | Hs.105465 |  |  | 1.1621 | 2.23783 | 3.551 | 12.9522 | 13.7968 | 10.5317 |
| 206695\_x\_at | ZNF43 | zinc finger protein 43 (HTF6) |  | NM\_003423 | 7594 | Hs.419763 | DNA binding  regulation of transcription, DNA-dependent  nucleus |  | 1.15057 | 2.22002 | 3.09333 | 12.1452 | 9.02377 | 9.68498 |
| 201316\_at | PSMA2 | proteasome (prosome, macropain) subunit, alpha type, 2 |  | AL523904 | 5683 | Hs.333786 |  | Proteasome | 1.13962 | 2.20324 | 4.01941 | 12.1164 | 12.5656 | 10.8483 |
| 203554\_x\_at | PTTG1 | pituitary tumor-transforming 1 |  | NM\_004219 | 9232 | Hs.350966 | cysteine protease inhibitor activity  protein binding  molecular\_function unknown  transcription factor activity  chromosome segregation  cell growth and/or maintenance  mitosis  spermatogenesis  DNA repair  transcription from Pol II promoter  cytoplasm  nucleus  cellular\_component unknown | Cell cycle | 1.13515 | 2.19642 | 3.25386 | 12.815 | 12.4685 | 12.095 |
| 205881\_at | ZNF74 | zinc finger protein 74 (Cos52) |  | NM\_003426 | 7625 | Hs.127476 | RNA binding  DNA binding  regulation of transcription, DNA-dependent  development  nucleus |  | 1.12353 | 2.17879 | 4.31311 | 9.1802 | 8.80356 | 6.7436 |
| 212176\_at | C6orf111 | chromosome 6 open reading frame 111 | Weakly similar to a region of TGN51 trans-Golgi network glycoprotein | AA902326 | 25957 | Hs.414993 |  |  | 1.10786 | 2.15526 | 4.02695 | 8.92133 | 11.6225 | 8.94524 |
| 218209\_s\_at | P15RS | hypothetical protein FLJ10656 |  | NM\_018170 | 55197 | Hs.300906 | kinase activity |  | 1.10339 | 2.14859 | 4.48338 | 9.65296 | 9.92798 | 9.69428 |
| 205036\_at | LSM6 | LSM6 homolog, U6 small nuclear RNA associated (S. cerevisiae) |  | NM\_007080 | 11157 | Hs.149675 | pre-mRNA splicing factor activity  RNA binding  RNA splicing  small nucleolar ribonucleoprotein complex  small nuclear ribonucleoprotein complex  nuclear mRNA splicing, via spliceosome |  | 1.10004 | 2.1436 | 2.55181 | 11.2476 | 10.749 | 10.1358 |
| 208447\_s\_at | PRPS1 | phosphoribosyl pyrophosphate synthetase 1 |  | NM\_002764 | 5631 | Hs.56 |  | Pentose phosphate pathway  Purine metabolism | 1.09426 | 2.13503 | 2.61577 | 8.61393 | 11.4621 | 9.71442 |
| 212627\_s\_at | KIAA0116 | KIAA0116 protein |  | AL581473 | 23016 | Hs.254717 | exonuclease activity  3'-5' exoribonuclease activity  RNA binding  rRNA processing  RNA catabolism  nucleus  hydrolase activity  exosome (RNase complex) |  | 1.08047 | 2.11473 | 4.53891 | 10.1273 | 11.9183 | 8.72115 |
| 208644\_at | ADPRT | ADP-ribosyltransferase (NAD+; poly (ADP-ribose) polymerase) |  | M32721 | 142 | Hs.177766 |  |  | 1.07961 | 2.11347 | 2.62491 | 9.89323 | 13.2694 | 11.1853 |
| 213793\_s\_at | HOMER1 | homer homolog 1 (Drosophila) |  | BE550452 | 9456 | Hs.129051 |  |  | 1.06847 | 2.09721 | 4.3015 | 9.61119 | 8.86816 | 10.4291 |
| 218491\_s\_at | THY28 | thymocyte protein thy28 |  | NM\_014174 | 29087 | Hs.13645 |  |  | 1.0677 | 2.09608 | 2.6521 | 11.6004 | 11.5399 | 10.4843 |
| 202954\_at | UBE2C | ubiquitin-conjugating enzyme E2C |  | NM\_007019 | 11065 | Hs.93002 | ubiquitin conjugating enzyme activity  ubiquitin-protein ligase activity  cyclin catabolism  ubiquitin cycle  positive regulation of cell proliferation  cell cycle  mitosis  ubiquitin-dependent protein catabolism  ligase activity  cytokinesis | Ubiquitin mediated proteolysis | 1.06463 | 2.09164 | 5.17372 | 8.77941 | 12.4916 | 11.1505 |
| 207453\_s\_at | DNAJB5 | DnaJ (Hsp40) homolog, subfamily B, member 5 |  | NM\_012266 | 25822 | Hs.237506 | heat shock protein activity  response to stress  protein folding |  | 1.0597 | 2.0845 | 1.52384 | 8.51668 | 9.59946 | 4.29382 |
| 202396\_at | TCERG1 | transcription elongation regulator 1 (CA150) |  | NM\_006706 | 10915 | Hs.300052 | RNA polymerase II transcription factor activity  transcription coactivator activity  transcription from Pol II promoter  nucleus |  | 1.05109 | 2.0721 | 3.2522 | 9.3133 | 11.3403 | 10.5103 |
| 201387\_s\_at | UCHL1 | ubiquitin carboxyl-terminal esterase L1 (ubiquitin thiolesterase) |  | NM\_004181 | 7345 | Hs.76118 | ubiquitin-dependent protein catabolism  intracellular  ubiquitin thiolesterase activity  hydrolase activity | Neurodegenerative Disorders  Parkinson's disease | 1.03977 | 2.0559 | 2.78513 | 12.7452 | 12.0684 | 11.5524 |
| 208697\_s\_at | EIF3S6 | eukaryotic translation initiation factor 3, subunit 6 48kDa |  | BC000734 | 3646 | Hs.405590 |  |  | 1.03383 | 2.04746 | 4.40041 | 11.4779 | 8.74394 | 13.0658 |
| 218951\_s\_at | FLJ11323 | hypothetical protein FLJ11323 |  | NM\_018390 | 55344 | Hs.378766 | phospholipase C activity  intracellular signaling cascade  ornithine decarboxylase activator activity |  | 1.0303 | 2.04245 | 4.1107 | 8.86783 | 10.112 | 9.38985 |
| 202469\_s\_at | CPSF6 | cleavage and polyadenylation specific factor 6, 68kDa |  | AU149367 | 11052 | Hs.64542 | nucleic acid binding  RNA binding  mRNA processing  nucleus |  | 1.01922 | 2.02683 | 3.64797 | 9.70929 | 10.4062 | 8.93426 |
| 200014\_s\_at | HNRPC | heterogeneous nuclear ribonucleoprotein C (C1/C2) |  | NM\_004500 | 3183 | Hs.476302 | RNA binding  RNA splicing  heterogeneous nuclear ribonucleoprotein complex |  | 1.01463 | 2.02039 | 3.38867 | 10.7996 | 10.793 | 10.912 |
| 201955\_at | CCNC | cyclin C |  | AL137784 | 892 | Hs.435450 | regulation of transcription, DNA-dependent  regulation of cell cycle  nucleus  cytokinesis |  | 1.01208 | 2.01682 | 3.15227 | 11.3144 | 10.6835 | 11.8869 |
| 219590\_x\_at | CGI-30 | CGI-30 protein |  | NM\_015958 | 51611 | Hs.440776 | methyltransferase activity  metabolism  diphthine synthase activity  transferase activity  peptidyl-diphthamide biosynthesis from peptidyl-histidine |  | 1.00381 | 2.00528 | 3.05352 | 10.2447 | 9.9355 | 8.11901 |
| 213762\_x\_at | RBMX | RNA binding motif protein, X-linked |  | AI452524 | 27316 | Hs.380118 | RNA binding  biological\_process unknown  heterogeneous nuclear ribonucleoprotein complex |  | 0.999092 | 1.99874 | 2.60966 | 11.7075 | 11.3844 | 12.305 |
| 218883\_s\_at | KLIP1 | KSHV latent nuclear antigen interacting protein 1 |  | NM\_024629 | 79682 | Hs.38178 |  |  | 0.99865 | 1.99813 | 2.96921 | 11.868 | 11.5411 | 11.2388 |
| 200826\_at | SNRPD2 | small nuclear ribonucleoprotein D2 polypeptide 16.5kDa |  | NM\_004597 | 6633 | Hs.424327 | pre-mRNA splicing factor activity  RNA splicing  spliceosome assembly  small nucleolar ribonucleoprotein complex  spliceosome complex  small nuclear ribonucleoprotein complex |  | 0.997682 | 1.99679 | 3.98123 | 10.7517 | 11.4811 | 13.388 |
| 203082\_at | BMS1L | BMS1-like, ribosome assembly protein (yeast) |  | NM\_014753 | 9790 | Hs.10848 | ATP binding  ribosome biogenesis  nucleus |  | 0.99469 | 1.99265 | 2.79397 | 10.7088 | 10.8964 | 9.41554 |
| 218319\_at | PELI1 | pellino homolog 1 (Drosophila) |  | NM\_020651 | 57162 | Hs.7886 |  |  | 0.991813 | 1.98868 | 1.72919 | 9.88283 | 11.3947 | 9.40594 |
| 206085\_s\_at | CTH | cystathionase (cystathionine gamma-lyase) |  | NM\_001902 | 1491 | Hs.19904 | amino acid metabolism  cystathionine gamma-lyase activity  lyase activity  cysteine biosynthesis | Methionine metabolism  Cysteine metabolism  Selenoamino acid metabolism  Nitrogen metabolism | 0.989575 | 1.9856 | 2.6891 | 8.98827 | 10.8077 | 7.13104 |
| 212317\_at | TNPO3 | transportin 3 |  | AK022910 | 23534 | Hs.412527 | receptor activity |  | 0.976477 | 1.96765 | 4.7313 | 10.784 | 11.3652 | 8.88148 |
| 218133\_s\_at | NIF3L1 | NIF3 NGG1 interacting factor 3-like 1 (S. pombe) |  | NM\_021824 | 60491 | Hs.21943 |  |  | 0.953107 | 1.93604 | 2.50444 | 10.685 | 10.9707 | 9.90832 |
| 209394\_at | ASMTL | acetylserotonin O-methyltransferase-like |  | BC002508 | 8623 | Hs.458420 | cellular\_component unknown  acetylserotonin O-methyltransferase activity  melatonin biosynthesis |  | 0.939617 | 1.91802 | 2.78688 | 9.16657 | 11.0078 | 9.926 |
| 201725\_at | C10orf7 | chromosome 10 open reading frame 7 | Strongly similar to rat cell cycle progression related D123 protein; has a putative role in cell cycle progression | NM\_006023 | 8872 | Hs.412842 | cell cycle arrest  positive regulation of cell proliferation |  | 0.923551 | 1.89678 | 2.97211 | 9.68045 | 12.1228 | 11.058 |
| 219617\_at | FLJ23451 | hypothetical protein FLJ23451 |  | NM\_024766 | 79823 | Hs.132799 |  |  | 0.901753 | 1.86833 | 3.24948 | 9.43309 | 8.47276 | 7.06567 |
| 202188\_at | KIAA0095 | KIAA0095 gene product |  | NM\_014669 | 9688 | Hs.295014 |  |  | 0.886339 | 1.84848 | 2.56032 | 11.6999 | 11.982 | 10.1197 |
| 218865\_at | FLJ22390 | hypothetical protein FLJ22390 |  | NM\_022746 | 64757 | Hs.195345 |  |  | 0.879979 | 1.84035 | 2.35903 | 7.7453 | 10.8336 | 8.02706 |
| 212145\_at | MRPS27 | mitochondrial ribosomal protein S27 |  | D87453 | 23107 | Hs.376200 | structural constituent of ribosome  mitochondrion |  | 0.879131 | 1.83927 | 2.09928 | 9.81803 | 11.3482 | 10.0552 |
| 208776\_at | PSMD11 | proteasome (prosome, macropain) 26S subunit, non-ATPase, 11 |  | BF432873 | 5717 | Hs.443379 | cytosol | Proteasome | 0.86428 | 1.82043 | 1.31914 | 11.2012 | 11.329 | 8.84049 |
| 217872\_at | FLJ20643 | hypothetical protein FLJ20643 |  | NM\_017916 | 55011 | Hs.5245 |  |  | 0.852679 | 1.80585 | 1.71395 | 10.3372 | 11.5679 | 9.39363 |
| 217964\_at | FLJ20343 | hypothetical protein FLJ20343 |  | NM\_017775 | 54902 | Hs.171044 |  |  | 0.824531 | 1.77096 | 2.15401 | 9.56602 | 11.7114 | 9.75573 |
| 202209\_at | LSM3 | LSM3 homolog, U6 small nuclear RNA associated (S. cerevisiae) |  | NM\_014463 | 27258 | Hs.111632 |  |  | 0.810107 | 1.75334 | 1.09003 | 10.529 | 12.7495 | 10.8957 |
| 219806\_s\_at | FN5 | FN5 protein |  | NM\_020179 | 56935 | Hs.416456 | molecular\_function unknown  biological\_process unknown  cellular\_component unknown |  | 0.789979 | 1.72905 | 2.42782 | 10.2813 | 9.88987 | 8.96336 |
| 218905\_at | FLJ20530 | hypothetical protein FLJ20530 |  | NM\_017864 | 55656 | Hs.370888 |  |  | 0.773114 | 1.70895 | 1.53013 | 10.0451 | 8.24735 | 9.70395 |
| 205661\_s\_at | PP591 | FAD-synthetase |  | NM\_025207 | 80308 | Hs.118666 | Mo-molybdopterin cofactor biosynthesis  metabolism  transferase activity |  | 0.760642 | 1.69424 | 1.42671 | 9.97581 | 10.7525 | 8.15418 |
| 200962\_at | RPL31 | ribosomal protein L31 |  | AI348010 | 6160 | Hs.375921 | structural constituent of ribosome  RNA binding  protein biosynthesis  cytosolic large ribosomal subunit (sensu Eukarya)  ribosome  intracellular | Ribosome | 0.719733 | 1.64688 | 1.76583 | 13.6898 | 14.1431 | 9.12239 |
| 210149\_s\_at | ATP5H | ATP synthase, H+ transporting, mitochondrial F0 complex, subunit d |  | AF061735 | 10476 | Hs.155728 |  | Oxidative phosphorylation  ATP synthesis | 0.711605 | 1.63763 | 0.946768 | 10.9627 | 12.1018 | 12.7069 |
| 205598\_at | TRIP | TRAF interacting protein |  | NM\_005879 | 10293 | Hs.21254 |  |  | 0.669013 | 1.58999 | 0.583055 | 9.40471 | 9.82539 | 5.30542 |

111 Genes
