# Supplementary material for: Comparing independent microarray studies: the case of human embryonic stem cells
Source: BMC Genomics. 2005 Jul 22;6:99. doi: 10.1186/1471-2164-6-99 (PMC1183205; doi:10.1186/1471-2164-6-99)
Supplement: Additional File 3 — Down-regulated genes in the intersection. List of down-regulated genes in the intersection of the 3 studies. In html format, including annotations and links. [file 1471-2164-6-99-S3.html]

Coherent and Significantly DOWN-regulated Genes across all studies


# Coherent and Significantly DOWN-regulated Genes across all studies

| Probe | Symbol | Description | Function | GenBank | LocusLink | UniGene | Gene Ontology | Pathway | Mmean | FCHmean | Bmean | ES-Bhatt | ES-Sperger | ES-Sato |
| --- | --- | --- | --- | --- | --- | --- | --- | --- | --- | --- | --- | --- | --- | --- |
| 202333\_s\_at | UBE2B | ubiquitin-conjugating enzyme E2B (RAD6 homolog) |  | AA877765 | 7320 | Hs.385986 | ubiquitin conjugating enzyme activity  ubiquitin-protein ligase activity  postreplication repair  ubiquitin cycle  ubiquitin-dependent protein catabolism  nucleus  ligase activity |  | -0.660179 | 0.6328 | 1.59915 | 9.11759 | 10.66 | 6.59747 |
| 200714\_x\_at | OS-9 | amplified in osteosarcoma |  | NM\_006812 | 10956 | Hs.76228 |  |  | -0.674826 | 0.626408 | 0.715796 | 11.4941 | 9.26429 | 8.29567 |
| 203943\_at | KIF3B | kinesin family member 3B |  | NM\_004798 | 9371 | Hs.301206 | plus-end-directed microtubule motor activity  ATP binding  anterograde axon cargo transport  determination of left/right symmetry  plus-end kinesin complex  microtubule associated complex |  | -0.781644 | 0.581703 | 1.45333 | 8.31978 | 10.0895 | 6.26489 |
| 209050\_s\_at | RALGDS | ral guanine nucleotide dissociation stimulator |  | AI421559 | 5900 | Hs.106185 | guanyl-nucleotide exchange factor activity  small GTPase mediated signal transduction  neuropeptide signaling pathway  intracellular signaling cascade  cellular\_component unknown |  | -0.797113 | 0.5755 | 1.79865 | 9.0853 | 8.9144 | 4.90455 |
| 213311\_s\_at | KIAA1049 | KIAA1049 protein |  | BF000251 | 22980 | Hs.227835 |  |  | -0.870914 | 0.5468 | 1.99522 | 11.1953 | 8.70546 | 8.04138 |
| 207000\_s\_at | PPP3CC | protein phosphatase 3 (formerly 2B), catalytic subunit, gamma isoform (calcineurin A gamma) |  | NM\_005605 | 5533 | Hs.75206 | phosphoprotein phosphatase activity  hydrolase activity  manganese ion binding |  | -0.881697 | 0.542729 | 2.14991 | 7.53844 | 8.8053 | 3.97559 |
| 202336\_s\_at | PAM | peptidylglycine alpha-amidating monooxygenase |  | NM\_000919 | 5066 | Hs.352733 | peptidylglycine monooxygenase activity  peptide amidation  secretory granule |  | -0.918234 | 0.529156 | 2.63483 | 9.6698 | 10.5435 | 7.52049 |
| 212715\_s\_at | KIAA0819 | KIAA0819 protein |  | AB020626 | 23289 | Hs.360864 |  |  | -0.96265 | 0.513114 | 2.52148 | 8.24561 | 8.07618 | 4.33318 |
| 221687\_s\_at | C9orf28 | chromosome 9 open reading frame 28 |  | BC000122 | 89853 | Hs.438972 |  |  | -0.982839 | 0.505983 | 2.80458 | 7.41336 | 6.16704 | 3.64505 |
| 203329\_at | PTPRM | protein tyrosine phosphatase, receptor type, M |  | NM\_002845 | 5797 | Hs.154151 | transmembrane receptor protein tyrosine phosphatase activity  receptor activity  protein amino acid dephosphorylation  integral to plasma membrane  hydrolase activity | Phosphatidylinositol signaling system | -1.05426 | 0.481544 | 3.80925 | 8.89187 | 8.69581 | 3.54874 |
| 203140\_at | BCL6 | B-cell CLL/lymphoma 6 (zinc finger protein 51) |  | NM\_001706 | 604 | Hs.155024 | protein binding  transcription factor activity  cell growth and/or maintenance  positive regulation of cell proliferation  regulation of transcription, DNA-dependent  inflammatory response  negative regulation of transcription from Pol II promoter  nucleus  mediator complex |  | -1.09321 | 0.468718 | 2.22008 | 7.87523 | 7.9755 | 4.49608 |
| 209420\_s\_at | SMPD1 | sphingomyelin phosphodiesterase 1, acid lysosomal (acid sphingomyelinase) |  | M59916 | 6609 | Hs.77813 | sphingomyelin phosphodiesterase activity  sphingomyelin metabolism  carbohydrate metabolism  neurogenesis  signal transduction  lysosome  hydrolase activity, acting on glycosyl bonds  integrase activity | Sphingophospholipid biosynthesis | -1.09698 | 0.467494 | 4.18931 | 8.23884 | 8.96717 | 4.39393 |
| 202191\_s\_at | GAS7 | growth arrest-specific 7 |  | BE439987 | 8522 | Hs.226133 | transcription factor activity  cell cycle arrest  cell growth and/or maintenance  development  neurogenesis |  | -1.11146 | 0.462825 | 3.67892 | 8.04257 | 12.0214 | 4.50473 |
| 202181\_at | KIAA0247 | KIAA0247 |  | NM\_014734 | 9766 | Hs.82426 | integral to membrane |  | -1.11419 | 0.461952 | 2.93538 | 7.9262 | 9.99649 | 5.36578 |
| 212830\_at | EGFL5 | EGF-like-domain, multiple 5 |  | W68084 | 1955 | Hs.236216 |  |  | -1.11638 | 0.461249 | 5.07456 | 7.20501 | 8.05237 | 3.62159 |
| 203047\_at | STK10 | serine/threonine kinase 10 |  | NM\_005990 | 6793 | Hs.16134 | kinase activity |  | -1.13322 | 0.455897 | 2.72642 | 8.54362 | 9.06224 | 5.60432 |
| 219390\_at | FKBP14 | FK506 binding protein 14, 22 kDa |  | NM\_017946 | 55033 | Hs.390838 | peptidyl-prolyl cis-trans isomerase activity  calcium ion binding  protein folding  endoplasmic reticulum  isomerase activity |  | -1.13532 | 0.455233 | 1.69739 | 9.13023 | 8.51067 | 7.14786 |
| 213103\_at | STARD13 | START domain containing 13 |  | AA128023 | 90627 | Hs.369912 |  |  | -1.13568 | 0.455119 | 3.48502 | 7.6763 | 8.8236 | 5.33687 |
| 201474\_s\_at | ITGA3 | integrin, alpha 3 (antigen CD49C, alpha 3 subunit of VLA-3 receptor) |  | NM\_002204 | 3675 | Hs.265829 | protein binding  receptor activity  integrin-mediated signaling pathway  cell-matrix adhesion  integrin complex  integral to membrane |  | -1.14449 | 0.452348 | 4.5166 | 9.18738 | 10.9539 | 5.62749 |
| 218065\_s\_at | C11orf15 | chromosome 11 open reading frame 15 |  | NM\_020644 | 56674 | Hs.389439 | integral to membrane |  | -1.14506 | 0.45217 | 2.43152 | 10.4217 | 7.43234 | 7.68619 |
| 201536\_at | DUSP3 | dual specificity phosphatase 3 (vaccinia virus phosphatase VH1-related) |  | AL048503 | 1845 | Hs.181046 | protein tyrosine/serine/threonine phosphatase activity  protein-tyrosine-phosphatase activity  protein amino acid dephosphorylation  hydrolase activity | Phosphatidylinositol signaling system | -1.21788 | 0.429913 | 3.09476 | 10.3244 | 9.05346 | 5.93442 |
| 217751\_at | LOC51064 | glutathione S-transferase subunit 13 homolog |  | NM\_015917 | 51064 | Hs.390667 | glutathione transferase activity  mitochondrion  transferase activity  protein disulfide oxidoreductase activity  periplasmic space (sensu Gram-negative Bacteria) | Glutathione metabolism | -1.227 | 0.427204 | 3.77825 | 9.06052 | 8.78238 | 7.4317 |
| 202982\_s\_at | ZAP128 | peroxisomal long-chain acyl-coA thioesterase |  | NM\_006821 | 10965 | Hs.446685 | serine esterase activity  acyl-CoA metabolism  peroxisome  acyl-CoA thioesterase activity  hydrolase activity  palmitoyl-CoA hydrolase activity |  | -1.22955 | 0.42645 | 4.71666 | 8.37269 | 8.13077 | 5.73513 |
| 218266\_s\_at | FREQ | frequenin homolog (Drosophila) |  | NM\_014286 | 23413 | Hs.301760 | calcium ion binding  cell communication  Golgi apparatus  cellular\_component unknown |  | -1.23239 | 0.425612 | 2.91851 | 8.51849 | 8.82164 | 4.85731 |
| 206200\_s\_at | ANXA11 | annexin A11 |  | NM\_001157 | 311 | Hs.75510 | protein binding  calcium-dependent phospholipid binding  calcium ion binding  immune response  nuclear membrane  nucleoplasm  cytoplasm |  | -1.23968 | 0.423468 | 4.2876 | 11.2509 | 10.6215 | 7.03901 |
| 202032\_s\_at | MAN2A2 | mannosidase, alpha, class 2A, member 2 |  | NM\_006122 | 4122 | Hs.116459 | mannosyl-oligosaccharide 1,3-1,6-alpha-mannosidase activity  alpha-mannosidase activity  carbohydrate metabolism  Golgi membrane  integral to membrane  hydrolase activity, acting on glycosyl bonds | N-Glycans biosynthesis | -1.25053 | 0.420295 | 5.83172 | 9.33816 | 8.94173 | 6.79214 |
| 222138\_s\_at | WDR13 | WD repeat domain 13 |  | AF158978 | 64743 | Hs.12142 | nucleus |  | -1.27342 | 0.413677 | 3.26373 | 9.67698 | 9.97622 | 5.92279 |
| 219456\_s\_at | RIN3 | Ras and Rab interactor 3 |  | AW027923 | 79890 | Hs.413374 | GTPase activator activity  neuropeptide signaling pathway  endocytosis  intracellular signaling cascade  cellular\_component unknown  Ras interactor activity |  | -1.28106 | 0.411493 | 5.58989 | 7.70693 | 8.27035 | 2.55344 |
| 210879\_s\_at | GAF1 | gamma-SNAP-associated factor 1 |  | AF334812 | 26056 | Hs.24557 | protein binding  biological\_process unknown  mitochondrial outer membrane  gamma-tubulin binding |  | -1.28109 | 0.411486 | 5.85784 | 9.13288 | 9.41614 | 4.4635 |
| 201554\_x\_at | GYG | glycogenin |  | NM\_004130 | 2992 | Hs.174071 |  |  | -1.28714 | 0.409762 | 4.47637 | 10.2556 | 10.2158 | 9.43139 |
| 208112\_x\_at | EHD1 | EH-domain containing 1 |  | NM\_006795 | 10938 | Hs.155119 | ATP binding  calcium ion binding  DNA binding  molecular\_function unknown  biological\_process unknown  intracellular  cellular\_component unknown |  | -1.29658 | 0.40709 | 5.39847 | 9.78201 | 9.75883 | 6.11547 |
| 203835\_at | GARP | glycoprotein A repetitions predominant |  | NM\_005512 | 2615 | Hs.151641 | integral to plasma membrane |  | -1.31546 | 0.401797 | 3.8018 | 9.20254 | 8.16248 | 3.71661 |
| 203254\_s\_at | TLN1 | talin 1 |  | NM\_006289 | 7094 | Hs.375001 | actin binding  structural constituent of cytoskeleton  cytoskeletal anchoring  cell motility  cytoskeleton  focal adhesion | Integrin-mediated cell adhesion | -1.3189 | 0.400839 | 5.66235 | 9.75538 | 9.30002 | 6.52629 |
| 204640\_s\_at | SPOP | speckle-type POZ protein |  | NM\_003563 | 8405 | Hs.129951 | protein binding  mRNA processing  nucleus |  | -1.32654 | 0.398724 | 5.75201 | 9.52618 | 9.6562 | 7.14293 |
| 202132\_at | TAZ | transcriptional co-activator with PDZ-binding motif (TAZ) |  | AA081084 | 25937 | Hs.24341 | transcription coactivator activity  regulation of transcription, DNA-dependent  nucleus |  | -1.36702 | 0.387691 | 4.81637 | 8.83085 | 8.74495 | 6.53475 |
| 203411\_s\_at | LMNA | lamin A/C |  | NM\_005572 | 4000 | Hs.436441 | protein binding  structural molecule activity  muscle development  lamin filament  nucleus |  | -1.36932 | 0.387073 | 4.38381 | 10.0113 | 8.9804 | 7.00982 |
| 201580\_s\_at | DJ971N18.2 | hypothetical protein DJ971N18.2 |  | AL544094 | 56255 | Hs.169358 | electron transporter activity  electron transport  integral to membrane |  | -1.38559 | 0.382734 | 6.76752 | 8.17581 | 9.15845 | 6.99497 |
| 208611\_s\_at | SPTAN1 | spectrin, alpha, non-erythrocytic 1 (alpha-fodrin) |  | U83867 | 6709 | Hs.387905 | actin binding  calmodulin binding  calcium ion binding  structural constituent of cytoskeleton  spectrin  membrane  cytoskeleton  membrane fraction |  | -1.39427 | 0.380437 | 5.84898 | 9.30995 | 7.12483 | 7.87937 |
| 205410\_s\_at | ATP2B4 | ATPase, Ca++ transporting, plasma membrane 4 |  | NM\_001684 | 493 | Hs.343522 | ATP binding  calcium-transporting ATPase activity  calmodulin binding  calcium ion binding  cation transport  transport  calcium ion transport  metabolism  integral to plasma membrane  magnesium ion binding  hydrolase activity  hydrolase activity, acting on acid anhydrides, catalyzing transmembrane movement of substances |  | -1.40899 | 0.376576 | 3.64338 | 8.49741 | 11.2213 | 5.48897 |
| 203167\_at | TIMP2 | tissue inhibitor of metalloproteinase 2 |  | NM\_003255 | 7077 | Hs.6441 | metalloendopeptidase inhibitor activity  extracellular matrix |  | -1.41407 | 0.375252 | 4.18735 | 9.31432 | 9.64158 | 5.71513 |
| 212615\_at | BC022889 | hypothetical protein BC022889 |  | AI742305 | 29962 | Hs.119053 | integral to membrane |  | -1.41606 | 0.374733 | 3.22393 | 9.76306 | 7.69548 | 6.78828 |
| 211075\_s\_at | CD47 | CD47 antigen (Rh-related antigen, integrin-associated signal transducer) |  | Z25521 | 961 | Hs.446414 | protein binding  integrin-mediated signaling pathway  cell-matrix adhesion  integral to plasma membrane |  | -1.43643 | 0.36948 | 6.01391 | 9.20899 | 8.57514 | 5.86823 |
| 209505\_at | NR2F1 | nuclear receptor subfamily 2, group F, member 1 |  | AI951185 | 7025 | Hs.361748 | ligand-regulated transcription factor activity  steroid hormone receptor activity  transcription coactivator activity  transcription factor activity  regulation of transcription, DNA-dependent  signal transduction  nucleus |  | -1.44783 | 0.366572 | 4.20317 | 8.06623 | 8.59729 | 3.96246 |
| 217168\_s\_at | HERPUD1 | homocysteine-inducible, endoplasmic reticulum stress-inducible, ubiquitin-like domain member 1 |  | AF217990 | 9709 | Hs.146393 | molecular\_function unknown  endoplasmic reticulum membrane  integral to membrane  response to unfolded protein |  | -1.4546 | 0.364857 | 5.86019 | 10.2411 | 9.00798 | 8.462 |
| 218436\_at | SIL1 | endoplasmic reticulum chaperone SIL1, homolog of yeast |  | NM\_022464 | 64374 | Hs.297875 | chaperone activity  intracellular protein transport  protein folding  endoplasmic reticulum |  | -1.45893 | 0.363763 | 6.51509 | 9.20876 | 8.58723 | 3.70417 |
| 212845\_at | SAMD4 | sterile alpha motif domain containing 4 | May mediate protein-protein interactions; contains a SAM (sterile alpha motif) domain | AB028976 | 23034 | Hs.98259 |  |  | -1.48353 | 0.357612 | 5.38293 | 7.17588 | 8.01583 | 4.00602 |
| 203882\_at | ISGF3G | interferon-stimulated transcription factor 3, gamma 48kDa |  | NM\_006084 | 10379 | Hs.1706 | transcription factor activity  regulation of transcription, DNA-dependent  cell surface receptor linked signal transduction  immune response  transcription from Pol II promoter  cytoplasm  nucleus |  | -1.50155 | 0.353174 | 4.37794 | 8.67859 | 9.45935 | 5.53297 |
| 204733\_at | KLK6 | kallikrein 6 (neurosin, zyme) |  | NM\_002774 | 5653 | Hs.79361 | trypsin activity  tissue kallikrein activity  protein binding  response to wounding  central nervous system development  extracellular  cytoplasm  chymotrypsin activity  protein autoprocessing  hydrolase activity  myelination  regulation of cell differentiation  hormone metabolism  collagen catabolism  tissue regeneration  amyloid precursor protein metabolism |  | -1.54554 | 0.342567 | 2.23813 | 7.80033 | 9.02167 | 3.06943 |
| 208817\_at | COMT | catechol-O-methyltransferase |  | BC000419 | 1312 | Hs.240013 | catecholamine metabolism  microsome  soluble fraction  integral to membrane  magnesium ion binding  transferase activity  neurotransmitter catabolism  catechol O-methyltransferase activity | Tyrosine metabolism | -1.56787 | 0.337307 | 6.79415 | 7.96184 | 11.0589 | 7.36578 |
| 203828\_s\_at | NK4 | natural killer cell transcript 4 |  | NM\_004221 | 9235 | Hs.943 |  |  | -1.58207 | 0.334003 | 5.67138 | 8.30977 | 10.4062 | 5.10015 |
| 207390\_s\_at | SMTN | smoothelin |  | NM\_006932 | 6525 | Hs.149098 | structural constituent of muscle  actin binding  muscle development  smooth muscle contraction  actin cytoskeleton |  | -1.59195 | 0.331723 | 5.07397 | 8.85803 | 9.22787 | 4.89029 |
| 201842\_s\_at | EFEMP1 | EGF-containing fibulin-like extracellular matrix protein 1 |  | AI826799 | 2202 | Hs.76224 | calcium ion binding  vision  extracellular matrix |  | -1.59257 | 0.33158 | 5.30936 | 8.16027 | 9.59447 | 4.4757 |
| 202794\_at | INPP1 | inositol polyphosphate-1-phosphatase |  | NM\_002194 | 3628 | Hs.32309 | inositol-1,4-bisphosphate 1-phosphatase activity  inositol/phosphatidylinositol phosphatase activity  phosphate metabolism  signal transduction  hydrolase activity | Inositol phosphate metabolism  Phosphatidylinositol signaling system | -1.60824 | 0.327999 | 7.06357 | 8.10837 | 9.17777 | 5.44646 |
| 212848\_s\_at | C9orf3 | chromosome 9 open reading frame 3 |  | BG036668 | 84909 | Hs.412286 | proteolysis and peptidolysis  membrane alanyl aminopeptidase activity |  | -1.60862 | 0.327912 | 6.11044 | 8.00649 | 9.06056 | 4.10447 |
| 202555\_s\_at | MYLK | myosin, light polypeptide kinase |  | NM\_005965 | 4638 | Hs.386078 | myosin-light-chain kinase activity  ATP binding  protein serine/threonine kinase activity  calmodulin binding  signal transducer activity  protein amino acid phosphorylation  kinase activity  transferase activity |  | -1.69668 | 0.308495 | 4.89071 | 7.83998 | 10.3772 | 5.66025 |
| 218029\_at | FLJ13725 | hypothetical protein FLJ13725 |  | NM\_024519 | 79567 | Hs.152717 |  |  | -1.70753 | 0.306184 | 6.53642 | 8.70056 | 9.49412 | 6.8457 |
| 202150\_s\_at | NEDD9 | neural precursor cell expressed, developmentally down-regulated 9 |  | U64317 | 4739 | Hs.388589 | actin bundling activity  protein binding  integrin-mediated signaling pathway  mitosis  cytoskeleton organization and biogenesis  cell adhesion  signal transduction  regulation of cell cycle  perinuclear space  spindle  cytoskeleton  regulation of cell growth |  | -1.7736 | 0.292477 | 4.30585 | 7.86685 | 7.89936 | 5.4266 |
| 218648\_at | TORC3 | transducer of regulated cAMP response element-binding protein (CREB) 3 |  | NM\_022769 | 64784 | Hs.434956 |  |  | -1.7805 | 0.291083 | 6.06988 | 9.40002 | 8.77639 | 5.7079 |
| 203729\_at | EMP3 | epithelial membrane protein 3 |  | NM\_001425 | 2014 | Hs.9999 | negative regulation of cell proliferation  cell death  development  cell proliferation  integral to membrane  membrane fraction  cell growth |  | -1.80045 | 0.287085 | 6.79778 | 8.59773 | 10.6946 | 5.55027 |
| 203490\_at | ELF4 | E74-like factor 4 (ets domain transcription factor) |  | NM\_001421 | 2000 | Hs.151139 | transcription coactivator activity  transcription factor activity  regulation of transcription, DNA-dependent  transcription from Pol II promoter  nucleus |  | -1.85889 | 0.275688 | 7.50646 | 9.5143 | 7.74201 | 4.79641 |
| 205409\_at | FOSL2 | FOS-like antigen 2 |  | NM\_005253 | 2355 | Hs.301612 | transcription factor activity  cell death  regulation of transcription from Pol II promoter  nucleus |  | -1.86316 | 0.274874 | 4.00951 | 8.5204 | 8.33849 | 4.11065 |
| 201278\_at | DAB2 | disabled homolog 2, mitogen-responsive phosphoprotein (Drosophila) |  | N21202 | 1601 | Hs.81988 | cell proliferation |  | -1.92974 | 0.262477 | 5.32593 | 8.63451 | 8.50283 | 4.13614 |
| 203821\_at | DTR | diphtheria toxin receptor (heparin-binding epidermal growth factor-like growth factor) |  | NM\_001945 | 1839 | Hs.799 | epidermal growth factor receptor binding  heparin binding  growth factor activity  receptor activity  positive regulation of cell proliferation  muscle development  signal transduction  integral to plasma membrane  extracellular space |  | -1.9597 | 0.257083 | 6.40144 | 8.81031 | 9.50579 | 4.22295 |
| 211026\_s\_at | MGLL | monoglyceride lipase |  | BC006230 | 11343 | Hs.409826 |  |  | -2.04183 | 0.242856 | 6.81291 | 7.49627 | 9.29303 | 5.09148 |
| 201963\_at | ACSL1 | acyl-CoA synthetase long-chain family member 1 |  | NM\_021122 | 2180 | Hs.406678 | long-chain-fatty-acid-CoA ligase activity  fatty acid metabolism  metabolism  digestion  magnesium ion binding  ligase activity | Fatty acid metabolism | -2.04559 | 0.242223 | 7.86577 | 8.36003 | 8.14587 | 5.994 |
| 203231\_s\_at | SCA1 | spinocerebellar ataxia 1 (olivopontocerebellar ataxia 1, autosomal dominant, ataxin 1) |  | AW235612 | 6310 | Hs.434961 | RNA binding  cytoplasm  nucleus |  | -2.05042 | 0.241415 | 6.61055 | 7.79135 | 7.30246 | 4.2294 |
| 218109\_s\_at | FLJ14153 | hypothetical protein FLJ14153 |  | NM\_022736 | 64747 | Hs.7503 | integral to membrane |  | -2.06695 | 0.238664 | 7.13252 | 8.0847 | 9.45139 | 5.33586 |
| 200794\_x\_at | DAZAP2 | DAZ associated protein 2 |  | NM\_014764 | 9802 | Hs.369761 |  |  | -2.06851 | 0.238406 | 4.80595 | 10.1302 | 9.17759 | 7.76442 |
| 200923\_at | LGALS3BP | lectin, galactoside-binding, soluble, 3 binding protein |  | NM\_005567 | 3959 | Hs.79339 | scavenger receptor activity  protein binding  cellular defense response  signal transduction  membrane  extracellular space |  | -2.06928 | 0.238279 | 4.89768 | 10.2882 | 10.6584 | 6.79715 |
| 209610\_s\_at | SLC1A4 | solute carrier family 1 (glutamate/neutral amino acid transporter), member 4 |  | BF340083 | 6509 | Hs.323878 | neutral amino acid transporter activity  neutral amino acid transport  dicarboxylic acid transport  transport  integral to plasma membrane  membrane fraction  sodium:dicarboxylate symporter activity  symporter activity |  | -2.19556 | 0.218309 | 6.55527 | 7.64597 | 7.81039 | 3.41165 |
| 200911\_s\_at | TACC1 | transforming, acidic coiled-coil containing protein 1 |  | NM\_006283 | 6867 | Hs.279245 | nucleus |  | -2.20037 | 0.217581 | 8.99 | 9.08362 | 8.59483 | 5.76915 |
| 220974\_x\_at | BA108L7.2 | similar to rat tricarboxylate carrier-like protein |  | NM\_030971 | 81855 | Hs.283844 | cation transporter activity  cation transport  membrane |  | -2.20632 | 0.216686 | 6.16263 | 9.61533 | 8.81767 | 4.39115 |
| 200696\_s\_at | GSN | gelsolin (amyloidosis, Finnish type) |  | NM\_000177 | 2934 | Hs.446537 | barbed-end actin capping/severing activity  actin filament severing activity  calcium ion binding  cytosol  extracellular  actin cytoskeleton  actin filament polymerization |  | -2.20899 | 0.216285 | 3.14257 | 9.75693 | 10.908 | 5.18382 |
| 200632\_s\_at | NDRG1 | N-myc downstream regulated gene 1 |  | NM\_006096 | 10397 | Hs.318567 |  |  | -2.21574 | 0.215276 | 6.96744 | 9.47201 | 9.28362 | 5.10635 |
| 212570\_at | KIAA0830 | KIAA0830 protein |  | AL573201 | 23052 | Hs.167115 |  |  | -2.29983 | 0.203087 | 7.4755 | 8.55353 | 7.71229 | 3.712 |
| 203065\_s\_at | CAV1 | caveolin 1, caveolae protein, 22kDa |  | NM\_001753 | 857 | Hs.74034 | structural molecule activity  caveola  integral to plasma membrane | Integrin-mediated cell adhesion | -2.30794 | 0.201948 | 5.88808 | 10.1634 | 10.8196 | 7.97553 |
| 212188\_at | KCTD12 | potassium channel tetramerisation domain containing 12 |  | AA551075 | 115207 | Hs.109438 | voltage-gated potassium channel activity  potassium ion transport  membrane  voltage-gated potassium channel complex |  | -2.45329 | 0.182594 | 7.02689 | 9.32371 | 6.80298 | 5.5095 |
| 221541\_at | DKFZP434B044 | hypothetical protein DKFZp434B044 |  | AL136861 | 83716 | Hs.262958 | extracellular |  | -2.46793 | 0.18075 | 5.61768 | 8.39989 | 8.15168 | 6.02073 |
| 1598\_g\_at | GAS6 | growth arrest-specific 6 |  | L13720 | 2621 | Hs.437710 | receptor binding  calcium ion binding  cell proliferation  signal transduction  extracellular |  | -2.4724 | 0.180191 | 7.92399 | 8.68046 | 5.91672 | 5.07108 |
| 201169\_s\_at | BHLHB2 | basic helix-loop-helix domain containing, class B, 2 |  | BG326045 | 8553 | Hs.171825 | transcription factor activity  regulation of transcription, DNA-dependent  nucleus | Circadian rhythm | -2.53647 | 0.172364 | 3.33281 | 8.83136 | 9.35521 | 3.54114 |
| 200838\_at | CTSB | cathepsin B |  | NM\_001908 | 1508 | Hs.135226 | cathepsin B activity  proteolysis and peptidolysis  lysosome  intracellular  hydrolase activity |  | -2.55086 | 0.170654 | 9.41577 | 10.9322 | 10.4533 | 7.08156 |
| 211986\_at | AHNAK | AHNAK nucleoprotein (desmoyokin) |  | BG287862 | 195 | Hs.378738 | neurogenesis  nucleus |  | -2.58195 | 0.167015 | 5.41674 | 8.70626 | 7.5358 | 3.35961 |
| 210139\_s\_at | PMP22 | peripheral myelin protein 22 |  | L03203 | 5376 | Hs.372031 | mechanosensory behavior  negative regulation of cell proliferation  hearing  peripheral nervous system development  synaptic transmission  integral to plasma membrane  membrane fraction |  | -2.74836 | 0.14882 | 8.54205 | 10.1293 | 7.86241 | 4.3536 |
| 209119\_x\_at | NR2F2 | nuclear receptor subfamily 2, group F, member 2 |  | AV703465 | 7026 | Hs.347991 | ligand-regulated transcription factor activity  transcription corepressor activity  steroid hormone receptor activity  transcription factor activity  lipid metabolism  signal transduction  regulation of transcription from Pol II promoter  nucleus |  | -2.75598 | 0.148036 | 7.47221 | 7.58541 | 8.41757 | 4.47988 |
| 202506\_at | SSFA2 | sperm specific antigen 2 |  | NM\_006751 | 6744 | Hs.438599 |  |  | -2.88528 | 0.135346 | 7.01628 | 8.06437 | 6.56965 | 4.93288 |
| 221667\_s\_at | HSPB8 | heat shock 27kDa protein 8 |  | AF133207 | 26353 | Hs.111676 | protein serine/threonine kinase activity  heat shock protein activity  biological\_process unknown  cellular\_component unknown  transferase activity |  | -2.89992 | 0.133979 | 7.45891 | 9.23046 | 9.56096 | 4.24864 |
| 218175\_at | FLJ22471 | limkain beta 2 |  | NM\_025140 | 80212 | Hs.387266 |  |  | -3.0383 | 0.121726 | 7.75768 | 7.95443 | 8.55461 | 4.25401 |
| 202935\_s\_at | SOX9 | SRY (sex determining region Y)-box 9 (campomelic dysplasia, autosomal sex-reversal) |  | AI382146 | 6662 | Hs.2316 | specific RNA polymerase II transcription factor activity  DNA binding  cartilage condensation  regulation of transcription from Pol II promoter  nucleus |  | -3.23287 | 0.106368 | 9.52252 | 10.0646 | 9.51032 | 2.74917 |
| 200878\_at | EPAS1 | endothelial PAS domain protein 1 |  | AF052094 | 2034 | Hs.8136 | transcription coactivator activity  RNA polymerase II transcription factor activity, enhancer binding  signal transducer activity  regulation of transcription, DNA-dependent  development  signal transduction  transcription from Pol II promoter  nucleus  angiogenesis |  | -3.25278 | 0.10491 | 4.55597 | 8.00678 | 8.12134 | 3.84483 |
| 218309\_at | CaMKIINalpha | calcium/calmodulin-dependent protein kinase II |  | NM\_018584 | 55450 | Hs.197922 | kinase activity |  | -3.29647 | 0.101781 | 11.1861 | 9.89538 | 9.09479 | 3.64443 |
| 201288\_at | ARHGDIB | Rho GDP dissociation inhibitor (GDI) beta |  | NM\_001175 | 397 | Hs.292738 | Rho GDP-dissociation inhibitor activity  GTPase activator activity  negative regulation of cell adhesion  Rho protein signal transduction  development  immune response  cytoplasmic vesicle  actin cytoskeleton organization and biogenesis |  | -3.37208 | 0.0965837 | 8.80606 | 8.21117 | 9.55108 | 4.83786 |
| 200974\_at | ACTA2 | actin, alpha 2, smooth muscle, aorta |  | NM\_001613 | 59 | Hs.208641 | structural constituent of muscle  motor activity  structural constituent of cytoskeleton  muscle development  actin filament  striated muscle thin filament |  | -4.03527 | 0.0609905 | 8.27545 | 8.14486 | 12.0139 | 6.15674 |
| 202291\_s\_at | MGP | matrix Gla protein |  | NM\_000900 | 4256 | Hs.365706 | structural constituent of bone  extracellular matrix structural constituent  calcium ion binding  ossification  cartilage condensation  extracellular matrix |  | -4.09479 | 0.0585255 | 7.49572 | 7.91172 | 8.13266 | 2.72409 |
| 218723\_s\_at | RGC32 | response gene to complement 32 |  | NM\_014059 | 28984 | Hs.76640 | regulation of CDK activity  cytoplasm |  | -4.13551 | 0.0568966 | 11.9562 | 7.71233 | 7.20499 | 3.12154 |
| 201162\_at | IGFBP7 | insulin-like growth factor binding protein 7 |  | NM\_001553 | 3490 | Hs.435795 | insulin-like growth factor binding  negative regulation of cell proliferation  extracellular  regulation of cell growth |  | -4.77173 | 0.0366071 | 11.3387 | 10.686 | 9.50366 | 4.21294 |

95 Genes
