# Supplementary material for: Comparing independent microarray studies: the case of human embryonic stem cells
Source: BMC Genomics. 2005 Jul 22;6:99. doi: 10.1186/1471-2164-6-99 (PMC1183205; doi:10.1186/1471-2164-6-99)
Supplement: Additional File 6 — Top 50 incoherent genes. List of the top 50 genes with the most negative coherence score. [file 1471-2164-6-99-S6.html]

Top 50 Genes with negative coherence score


# Top 50 Genes with negative coherence score

| Probe | Symbol | Description | Function | GenBank | LocusLink | UniGene | Gene Ontology | Pathway | value |
| --- | --- | --- | --- | --- | --- | --- | --- | --- | --- |
| 208660\_at | CS | citrate synthase |  | BC000105 | 1431 | Hs.430606 | citrate (Si)-synthase activity  tricarboxylic acid cycle  mitochondrion  transferase activity | Citrate cycle (TCA cycle)  Glyoxylate and dicarboxylate metabolism | -0.15 |
| 218896\_s\_at | HSA277841 | ELG protein |  | NM\_018553 | 55421 | Hs.120963 |  |  | -0.1483 |
| 203636\_at | MID1 | midline 1 (Opitz/BBB syndrome) |  | BE967532 | 4281 | Hs.27695 | protein binding  zinc ion binding  morphogenesis  pattern specification  microtubule cytoskeleton organization and biogenesis  regulation of transcription, DNA-dependent  microtubule associated complex  nucleus |  | -0.1466 |
| 203934\_at | KDR | kinase insert domain receptor (a type III receptor tyrosine kinase) |  | NM\_002253 | 3791 | Hs.12337 | vascular endothelial growth factor receptor activity  ATP binding  receptor activity  transmembrane receptor protein tyrosine kinase signaling pathway  protein amino acid phosphorylation  integral to plasma membrane  angiogenesis  transferase activity |  | -0.1411 |
| 212065\_s\_at | USP34 | ubiquitin specific protease 34 |  | AW502434 | 9736 | Hs.507665 | cysteine-type endopeptidase activity  ubiquitin-dependent protein catabolism  ubiquitin thiolesterase activity |  | -0.1408 |
| 203536\_s\_at | CIAO1 | WD40 protein Ciao1 |  | NM\_004804 | 9391 | Hs.12109 | positive regulation of cell proliferation  regulation of transcription from Pol II promoter  nucleus |  | -0.1399 |
| 211071\_s\_at | AF1Q | ALL1-fused gene from chromosome 1q |  | BC006471 | 10962 | Hs.75823 | molecular\_function unknown  cell growth and/or maintenance  cellular\_component unknown |  | -0.1395 |
| 201701\_s\_at | PGRMC2 | progesterone receptor membrane component 2 |  | NM\_006320 | 10424 | Hs.9071 | steroid hormone receptor activity  steroid binding  receptor activity  microsome  integral to membrane |  | -0.1395 |
| 205945\_at | IL6R | interleukin 6 receptor |  | NM\_000565 | 3570 | Hs.193400 | interleukin-6 receptor activity  receptor activity  skeletal development  cell surface receptor linked signal transduction  development  immune response  cell proliferation  interleukin-6 receptor complex  integral to membrane  plasma membrane  hematopoietin/interferon-class (D200-domain) cytokine receptor activity |  | -0.1394 |
| 211177\_s\_at | TXNRD2 | thioredoxin reductase 2 |  | AB019695 | 10587 | Hs.443430 | thioredoxin-disulfide reductase activity  electron transport  mitochondrion  disulfide oxidoreductase activity  response to oxygen radicals |  | -0.1389 |
| 204552\_at | INPP4A | inositol polyphosphate-4-phosphatase, type I, 107kDa |  | AA355179 | 3631 | Hs.334575 | signal transduction | Inositol phosphate metabolism  Phosphatidylinositol signaling system | -0.1366 |
| 207069\_s\_at | MADH6 | MAD, mothers against decapentaplegic homolog 6 (Drosophila) |  | NM\_005585 | 4091 | Hs.153863 | receptor signaling protein serine/threonine kinase signaling protein activity  protein binding  signal transducer activity  regulation of transcription, DNA-dependent  intracellular  transforming growth factor beta receptor, inhibitory cytoplasmic mediator activity |  | -0.1358 |
| 201106\_at | GPX4 | glutathione peroxidase 4 (phospholipid hydroperoxidase) |  | NM\_002085 | 2879 | Hs.433951 | peroxidase activity  glutathione peroxidase activity  electron transporter activity  phospholipid metabolism  response to oxidative stress  development  mitochondrion  oxidoreductase activity | Glutathione metabolism | -0.1343 |
| 203358\_s\_at | EZH2 | enhancer of zeste homolog 2 (Drosophila) |  | NM\_004456 | 2146 | Hs.444082 | DNA binding  establishment and/or maintenance of chromatin architecture  regulation of transcription, DNA-dependent  nucleus |  | -0.1343 |
| 203525\_s\_at | APC | adenomatosis polyposis coli |  | AI375486 | 324 | Hs.75081 | beta-catenin binding  cell adhesion  protein complex assembly  signal transduction  Wnt receptor signaling pathway  negative regulation of cell cycle |  | -0.1343 |
| 217177\_s\_at | PTPRB | protein tyrosine phosphatase, receptor type, B |  | AL080103 | 5787 | Hs.434375 | transmembrane receptor protein tyrosine phosphatase activity  phosphate metabolism  protein amino acid dephosphorylation  integral to plasma membrane  membrane fraction  hydrolase activity | Phosphatidylinositol signaling system | -0.134 |
| 213932\_x\_at | HLA-A | major histocompatibility complex, class I, A |  | AI923492 | 3105 | Hs.181244 | immune response  integral to plasma membrane  antigen presentation, endogenous antigen  antigen processing, endogenous antigen via MHC class I  MHC class I receptor activity |  | -0.1339 |
| 221741\_s\_at | C20orf21 | chromosome 20 open reading frame 21 | Highly similar to a region of HGRG8 | AL096828 | 54915 | Hs.11747 |  |  | -0.1335 |
| 211944\_at | XTP2 | HBxAg transactivated protein 2 |  | BE729523 | 23215 | Hs.446197 |  |  | -0.1331 |
| 222133\_s\_at | CGI-72 | CGI-72 protein |  | AK022280 | 51105 | Hs.44159 | nucleic acid binding |  | -0.1328 |
| 214688\_at | TLE4 | transducin-like enhancer of split 4 (E(sp1) homolog, Drosophila) |  | BF217301 | 7091 | Hs.494269 | molecular\_function unknown  frizzled signaling pathway  regulation of transcription, DNA-dependent  biological\_process unknown  nucleus |  | -0.1315 |
| 203990\_s\_at | UTX | ubiquitously transcribed tetratricopeptide repeat gene, X chromosome |  | AI140752 | 7403 | Hs.201790 | nucleus |  | -0.1311 |
| 221059\_s\_at | CHST6 | carbohydrate (N-acetylglucosamine 6-O) sulfotransferase 6 |  | NM\_021615 | 4166 | Hs.157439 | N-acetylglucosamine metabolism  Golgi apparatus  transferase activity  N-acetylglucosamine 6-O-sulfotransferase activity |  | -0.1306 |
| 39402\_at | IL1B | interleukin 1, beta |  | M15330 | 3553 | Hs.126256 | interleukin-1 receptor binding  signal transducer activity  negative regulation of cell proliferation  apoptosis  immune response  cell proliferation  signal transduction  regulation of cell cycle  cell-cell signaling  inflammatory response  extracellular space  antimicrobial humoral response (sensu Vertebrata) |  | -0.1301 |
| 209980\_s\_at | SHMT1 | serine hydroxymethyltransferase 1 (soluble) |  | L23928 | 6470 | Hs.293636 | glycine metabolism  L-serine catabolism  cytosol  glycine hydroxymethyltransferase activity  one-carbon compound metabolism  transferase activity | Glycine, serine and threonine metabolism  Lysine degradation  Cyanoamino acid metabolism  One carbon pool by folate  Methane metabolism | -0.1298 |
| 204638\_at | ACP5 | acid phosphatase 5, tartrate resistant |  | NM\_001611 | 54 | Hs.1211 |  | gamma-Hexachlorocyclohexane degradation  Riboflavin metabolism | -0.1296 |
| 214724\_at | DIXDC1 | DIX domain containing 1 |  | AF070621 | 85458 | Hs.116796 | signal transducer activity  frizzled signaling pathway  development  intracellular |  | -0.1295 |
| 204324\_s\_at | GOLPH4 | golgi phosphoprotein 4 |  | NM\_014498 | 27333 | Hs.143600 | Golgi cis-face  Golgi lumen  integral to membrane  endocytic vesicle |  | -0.1292 |
| 207761\_s\_at | DKFZP586A0522 | DKFZP586A0522 protein |  | NM\_014033 | 25840 | Hs.288771 | S-adenosylmethionine-dependent methyltransferase activity |  | -0.129 |
| 202994\_s\_at | E46L | like mouse brain protein E46 |  | Z95331 | 25814 | Hs.191294 | molecular\_function unknown  biological\_process unknown  cellular\_component unknown |  | -0.1288 |
| 202974\_at | MPP1 | membrane protein, palmitoylated 1, 55kDa |  | NM\_002436 | 4354 | Hs.422215 | guanylate kinase activity  protein binding  signal transduction  membrane  integral to plasma membrane  membrane fraction |  | -0.1288 |
| 208621\_s\_at | VIL2 | villin 2 (ezrin) |  | BF663141 | 7430 | Hs.403997 | cytoskeleton |  | -0.1282 |
| 209083\_at | CORO1A | coronin, actin binding protein, 1A |  | U34690 | 11151 | Hs.415067 | actin binding  structural molecule activity  transport  phagosome formation  mitosis  cell motility  lysosomal membrane  actin cytoskeleton |  | -0.1282 |
| 205402\_x\_at | PRSS2 | protease, serine, 2 (trypsin 2) |  | NM\_002770 | 5645 | Hs.511525 | trypsin activity  calcium ion binding  proteolysis and peptidolysis  digestion  chymotrypsin activity  hydrolase activity |  | -0.1281 |
| 207540\_s\_at | SYK | spleen tyrosine kinase |  | NM\_003177 | 6850 | Hs.192182 | protein-tyrosine kinase activity  ATP binding  protein amino acid phosphorylation  intracellular signaling cascade  cell proliferation  protein complex assembly  organogenesis  transferase activity |  | -0.128 |
| 204082\_at | PBX3 | pre-B-cell leukemia transcription factor 3 |  | NM\_006195 | 5090 | Hs.294101 | protein binding  transcription factor activity  anterior compartment specification  posterior compartment specification  regulation of transcription, DNA-dependent  nucleus  embryonic development  ribulose bisphosphate carboxylase complex  carbon utilization by fixation of carbon dioxide  ribulose-bisphosphate carboxylase activity  regulation of transcriptional preinitiation complex formation |  | -0.1279 |
| 213631\_x\_at | DHODH | dihydroorotate dehydrogenase |  | BE789211 | 1723 | Hs.405639 | dihydroorotate dehydrogenase activity  'de novo' pyrimidine base biosynthesis  membrane  mitochondrion  mitochondrial inner membrane  pyrimidine nucleotide biosynthesis  oxidoreductase activity  dihydroorotate oxidase activity | Pyrimidine metabolism | -0.1279 |
| 220488\_s\_at | BCAS3 | breast carcinoma amplified sequence 3 |  | NM\_017679 | 54828 | Hs.287450 | cell growth and/or maintenance  nucleus |  | -0.1277 |
| 220419\_s\_at | USP25 | ubiquitin specific protease 25 |  | NM\_013396 | 29761 | Hs.186961 | ubiquitin-specific protease activity  cysteine-type endopeptidase activity  proteolysis and peptidolysis  ubiquitin-dependent protein catabolism  protein modification  ubiquitin thiolesterase activity |  | -0.1275 |
| 206404\_at | FGF9 | fibroblast growth factor 9 (glia-activating factor) |  | NM\_002010 | 2254 | Hs.111 | heparin binding  growth factor activity  cell proliferation  signal transduction  regulation of cell cycle  cell-cell signaling  extracellular space  cell differentiation |  | -0.1266 |
| 221098\_x\_at | SDCCAG16 | serologically defined colon cancer antigen 16 | Colon cancer antigen 16 | NM\_006649 | 10813 | Hs.271926 | tumor antigen |  | -0.1265 |
| 208868\_s\_at | GABARAPL1 | GABA(A) receptor-associated protein like 1 |  | BF125756 | 23710 | Hs.336429 |  |  | -0.1264 |
| 203256\_at | CDH3 | cadherin 3, type 1, P-cadherin (placental) |  | NM\_001793 | 1001 | Hs.191842 |  |  | -0.1257 |
| 218872\_at | TSC | hypothetical protein FLJ20607 |  | NM\_017899 | 54997 | Hs.345908 | calcium ion binding |  | -0.1251 |
| 204639\_at | ADA | adenosine deaminase |  | NM\_000022 | 100 | Hs.407135 | adenosine deaminase activity  nucleotide metabolism  hydrolase activity  antimicrobial humoral response (sensu Vertebrata)  purine ribonucleoside monophosphate biosynthesis | Purine metabolism | -0.1251 |
| 210198\_s\_at | PLP1 | proteolipid protein 1 (Pelizaeus-Merzbacher disease, spastic paraplegia 2, uncomplicated) |  | BC002665 | 5354 | Hs.1787 |  |  | -0.1241 |
| 204155\_s\_at | KIAA0999 | KIAA0999 protein |  | AA044154 | 23387 | Hs.444909 | ATP binding  protein serine/threonine kinase activity  protein amino acid phosphorylation  transferase activity |  | -0.124 |
| 218654\_s\_at | MRPS33 | mitochondrial ribosomal protein S33 |  | NM\_016071 | 51650 | Hs.83006 | structural constituent of ribosome  protein biosynthesis  mitochondrion  mitochondrial small ribosomal subunit |  | -0.1233 |
| 212275\_s\_at | SRCAP | Snf2-related CBP activator protein |  | NM\_006662 | 10847 | Hs.136227 | ATP binding  DNA binding  regulation of transcription, DNA-dependent  nucleus  ATP dependent helicase activity  hydrolase activity |  | -0.1227 |
| 200854\_at | NCOR1 | nuclear receptor co-repressor 1 |  | AB028970 | 9611 | Hs.144904 | transcription corepressor activity  DNA binding  regulation of transcription, DNA-dependent  transcription from Pol II promoter  nucleus | Huntington's disease | -0.1226 |

50 Genes
